# CXCL12/CXCR4 facilitates perineural invasion via induction of the Twist/S100A4 axis in salivary adenoid cystic carcinoma

**DOI:** 10.1111/jcmm.16713

**Published:** 2021-06-25

**Authors:** Mei Zhang, Min Zheng, Li Dai, Wei‐long Zhang, Hua‐yang Fan, Xiang‐hua Yu, Xin Pang, Peng Liao, Bing‐jun Chen, Sha‐sha Wang, Ming‐xin Cao, Xiang‐rui Ma, Xin‐hua Liang, Ya‐ling Tang

**Affiliations:** ^1^ State Key Laboratory of Oral Diseases & National Clinical Research Center for Oral Diseases & Department of Oral and Maxillofacial Surgery West China Hospital of Stomatology (Sichuan University) Chengdu China; ^2^ Department of Stomatology Zhoushan Hospital Wenzhou Medical University. Zhoushan Zhejiang China; ^3^ State Key Laboratory of Oral Diseases & National Clinical Research Center for Oral Diseases & Department of Oral Pathology West China Hospital of Stomatology (Sichuan University) Chengdu China; ^4^ Department of Oral and Maxillofacial Surgery Binzhou Medical University Hospital Binzhou China

**Keywords:** CXCL12/CXCR4, perineural invasion (PNI), salivary adenoid cystic carcinoma (SACC), Schwann cell

## Abstract

The activation of CXCL12/CXCR4 axis participated in the progression of multiple cancers, but potential effect in terms of perineural invasion (PNI) in SACC remained ambiguous. In this study, we identified that CXCL12 substantially expressed in nerve cells. CXCR4 strikingly expressed in tumour cells, and CXCR4 expression was closely associated with the level of EMT‐associated proteins and Schwann cell hallmarks at nerve invasion frontier in SACC. Activation of CXCL12/CXCR4 axis could promote PNI and up‐regulate relative genes of EMT and Schwann cell hallmarks both in vitro and in vivo, which could be inhibited by Twist silence. After overexpressing S100A4, the impaired PNI ability of SACC cells induced by Twist knockdown was significantly reversed, and pseudo foot was visualized frequently. Collectively, the results indicated that CXCL12/CXCR4 might promote PNI by provoking the tumour cell to differentiate towards Schwann‐like cell through Twist/S100A4 axis in SACC.

## 1 INTRODUCTION

Salivary adenoid cystic carcinoma (SACC) constitutes a quarter of malignant neoplasms derived from salivary gland. Despite slow indolent growth and the 5‐year overall survival rate of patients with SACC generally up to 90%, it dropped to 10% in two decades because of insidious local recurrence and haematogenous metastasis.^1^ Perineural invasion (PNI), recognized as tumour cells disseminating along nerves,^2^ is a prominent histological characteristic in SACC, impedes surgical treatment and indicates a decreased survival rate.^3^, ^4^, ^5^ Therefore, unveiling latent intertwining underlying PNI might advocate novel treatment for SACC patients with PNI.

The role of CXCR4, who functions through combing its cognate chemokine ligand CXCL12 to orchestrate cancer cells directly or through inducing angiogenesis and recruiting immune cells indirectly, is broadly concerned in multiple tumour‐promoting processes.^6^, ^7^ Evidence revealed that the activation of CXCL12/CXCR4 could promote invasion and metastasis in breast cancer,^8^ gastric cancer ^9^ and glioblastoma.^10^ However, whether the activation of CXCL12/CXCR4 signalling axis can promote PNI of SACC is not yet fully clear.

Schwann cells are fundamental to the merisis and luxuriance of nerves and conducive to the guidance of axons.^11^, ^12^ A growing body of evidence showed that the level of Schwann cell hallmarks, including glial fibrillary acidic protein (GFAP), p75 neurotrophin receptor (p75NTR), S100 calcium‐binding protein A4 (S100A4), was significantly increased in many tumour with PNI, including melanoma,^13^ breast cancer,^14^ prostate cancer,^15^ colorectal cancer.^16^ Hence, researchers inferred that the differentiation of cancer cell towards Schwann‐like cell might be responsible for the PNI occurrence of tumours.^17^, ^18^ In SACC species, the level of GFAP and S100A4 has been reported to be dramatically elevated at frontier of nerve invasion.^19^ However, the relevance between CXCL12/CXCR4 and the differentiation of tumour cells towards Schwann‐like cell in PNI of SACC has not been investigated.

Thus, our purpose was to examine the expression of CXCL12/CXCR4 in 158 SACC species and assess the relation of CXCL12/CXCR4 expression with patients’ clinicopathological factors, notably PNI. Then, we further explored whether the activation of CXCL12/CXCR4 signalling was an orchestrator of EMT and contributed to the migration, invasion and PNI through promoting SACC cells to differentiate towards Schwann‐like cell. Clarifying the molecular mechanism of CXCL12/CXCR4 regulating PNI in SACC will be helpful to the development of molecule‐targeted treatment and eradication of relapses induced by the PNI in SACC.

## 2 MATERIALS AND METHODS

### 2.1 Patients and specimens

Sections of 158 SACC patients by surgical resection with no chemotherapy, radiotherapy or hormone therapy prior to surgery and 20 precancerous glands, histologically confirmed by two pathologists blindly, were selected with informed consents from 2002 to 2007 in West China Hospital of Stomatology, Sichuan University. EthSical approval of present study was granted by the Institutional Ethics Committee of West China Medical Center, Sichuan University. The follow‐up information was renewed on 31 December 2017, and median follow‐up was 83.7 months (3.5‐140 months).

### 2.2 Immunohistochemistry

Embedded sections were performed according to conventional immunohistochemistry procedure. Primary antibodies from Abcam were applied for immunostaining. The semi‐quantitative assessment of immunohistochemical staining was determined by positive cell ratio in 1000 cells randomly at 200× magnification, and six microscopic fields were observed per section. The intensity was classified as 0 (−, negative), <5%; 1 (+, weak), 5%‐25%; 2 (++, moderate), 25%‐50%; 3 (+++, strong), >50%. 2 and 3 indicated high expression, and 0 and 1 indicated low expression.

### 2.3 Cell line and cell culture

SACC cell lines (SACC‐83 and SACC‐LM) and nerve derived cell microglia BV2 (State Key Laboratory of Oral Diseases & National Clinical Research Center for Oral Diseases, Sichuan University) were enrolled and suspended in DMEM (HyClone) medium with 10% foetal bovine serum (Invitrogen) and 1% streptomycin/penicillin in incubator containing 5% CO_2_ at 37℃.

### 2.4 Cell transfection

To knockdown CXCR4 and Twist of SACC cells, siRNA targeting CXCR4 and Twist was synthesized and transfected into SACC cells with Lipofectamine 2000 reagent (Invitrogen). S100A4 was integrated into pcDNA3.1 (Invitrogen) to overexpress S100A4 following the guidelines of protocol.

### 2.5 Immunofluorescence staining

Cells reaching 75% in 24‐well plates were immobilized in 4% paraformaldehyde and permeabilized by PBS containing 0.25% Triton X‐100. Primary antibody was incubated at 4℃ overnight after blocking using normal goat serum. Following day, the cells were subjected to counterstain with 4', 6‐diamidino‐2‐phenylindole (DAPI) and viewed via a fluorescence microscope.

### 2.6 Quantitative real‐time PCR (qRT‐PCR)

Total RNA in cells and tissues was isolated via TRIzol reagent (Takara, Tokyo) and then transcribed into cDNA after the purity and concentration of RNA were guaranteed with the NanoDrop^®^ ND‐1000 Spectrophotometer (Thermo Fisher). QRT‐PCR was accomplished according to the protocol.

### 2.7 Scratch wound healing assay

Cells were cultured, and a scratch was created gently via a 200 μL sterile pipette tip once reaching approximately 100% cell confluence. Detached cells and debris were eliminated through PBS rewashing. Thereupon, SACC cells were incubated within medium excluded serum and scratches were photographed. Percentage of occupied area by migratory cells was quantified by Image Analysis software.

### 2.8 Transwell invasion assay

Approximately 1 × 10^5^ SACC cells were resuspended into the upper chamber of the Corning Matrigel Invasion insert covered with diluted Matrigel (BD Biosciences) filled with serum‐free medium given 48 hours to invade. The insert was placed in 24‐well culture plate containing medium supplemented with 10% FBS. Non‐invading cells remaining on the surfaces of upper chamber were scraped off. Invaded cells were stained and counted in five separate fields per well, randomly.

### 2.9 In vitro nerve and tumour cells co‐culture assay

For the investigation of the CXCL12/CXCR4 signalling axis on the neurophilic invasion behaviour of SACC cells, we adjusted the nerve and tumour cells in vitro co‐culture model in accordance with the method preveniently established by researchers.^20^, ^21^, ^22^ Dorsal root ganglia (DRG) of newborn rat was dissected and embedded in the centre of the 6‐well plate containing 20 μL of Matrigel. SACC cells (10^4^) were suspended beyond DRG and the following day was logged as 1 day. Nerve and tumour cell co‐culture system was examined at 1 day and 3 day, respectively, and images were collected. To analyse the results quantitatively, the least range between the DRG and SACC cells was delimited. PNI activity was resolved by the quantity of migratory tumour cells around DRG within least gap within random five fields in three independent experiments using Image Analysis software.

### 2.10 Phalloidin staining

SACC cell was fixed with paraformaldehyde, permeabilized using PBS containing Triton X‐100, blocked in BSA and stained with TRITC‐phalloidin (Sigma), testing for cytoskeletal reorganization at room temperature. Then, DAPI was employed to stain the nucleus. Images were viewed using fluorescence microscopy.

### 2.11 In vivo PNI model

Twenty nude mice (5 weeks old) were obtained to establish the PNI model of SACC after adapting to the environment for a week. The mouse was anaesthetized and received groin injection of 2.5 × 10^6^ SACC‐LM cells. Two weeks after inoculation, 20 nude mice were randomized allocated into three group, including saline group (n = 6, treatment with saline at 0.1 mL/d), AMD3100 group (CXCR4 antagonist) (n = 7, treatment with AMD3100 at 2 mg/kg) and CXCL12 group (n = 7, treatment with CXCL12 at 5 μg/kg),^23^ and received corresponding treatment for 2 weeks, respectively. Sciatic nerve function, which intervened functional movement of hind limbs in nude mice, was observed and recorded weekly, and observation indicators include the following: (a) sciatic nerve function index, evaluated by measuring the reach distant (mm) of the first toe to fifth toe of the nude mice’ hind claw, (b) function of hind limb, which was classified from 4 (normal) to 1 (total paw paralysis) based on hind paw’ action to the manually stretching of the soma. Samples were taken, and subsequent experiments were conducted after 7 weeks.^24^, ^25^

### 2.12 Statistical analysis

Statistical analysis was carried out via SPSS Version 21.0 (IBM Corp). The level of proteins and patients’ clinicopathologic features were evaluated by Wilcoxon test. Correlation coefficient of two variables was calculated using 2‐tailed Pearson's statistics. *P* < .05 was considered to be statistically significant, and multi‐groups differences were corrected using Bonferroni.

## 3 RESULTS

### 3.1 Relationship between CXCL12/CXCR4 and PNI in human SACC specimens

**FIGURE 1:**
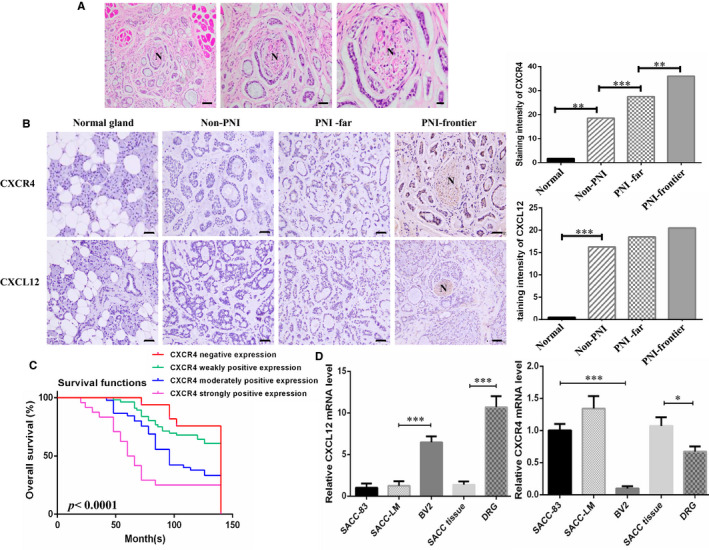
CXCL12/CXCR4 associated with the PNI in SACC patients. A, HE staining showed that nerve was encroached by tumour cells in SACC patients. Representational results are displayed. Magnification: Left 100×, Mid 200×, Right 400×. B, Immunohistochemical staining of CXCL12 and CXCR4 in different tissue. ‘Normal gland’ indicates normal salivary gland. ‘Non‐PNI’ indicates SACC without PNI. ‘PNI‐far’ indicates SACC far away nerve site. ‘PNI‐frontier’ indicates nerve invasion frontier. SACC at nerve invasion front displayed higher expression of CXCR4. CXCL12 was mainly expressed by nerve tissue, (200×). C, Kaplan‐Meier analysis for SACC patients’ overall survival time with differential level of CXCR4 was conducted. D, qRT‐PCR analyses of CXCL12 and CXCR4 mRNA level in SACC cell lines (SACC‐83 and SACC‐LM), SACC tissue, nerve cell BV2 and DRG tissue. *P < .05, ***P < .001

To explore the significance of CXCL12/CXCR4 in human SACC tissues, immunohistochemical staining was applied in 158 cases of SACC, in which there were 77 cases (48.7%) with PNI (Figure 1A). CXCR4, generally stained in cytoplasm, was overexpressed in 79.1% (125/158) SACC specimens, whereas weak positive expression in 35% (7/20) normal salivary gland (Figure 1B). CXCL12, stained in cytoplasm, was mainly expressed in nerve cells and occasionally detected in tumour cells of 51.9% (82/158) SACC specimens, and 10% (2/20) in normal salivary gland was weak expression (Figure 1B). The level of CXCR4 and CXCL12 in SACC was higher relative to normal gland (*P* = .005, *P* = .001). The relevance between CXCR4 level and clinicopathologic features showed that CXCR4 level was noticeably associated with tumour location, clinical staging, histological subtype, implication of resection margin, relapse and metastasis (*P* < .05, Table 1). CXCL12 expression in SACC was rarely linked to any clinicopathologic features (*P* > .05).

**TABLE 1:** Correlation between CXCR4 expression and clinicopathological parameters of SACC patients

Clinicopathological parameters	Cases	CXCR4 expression				*P* value
		‐	+	++	+++	
	158	33	56	45	24	
Ages (years)						
<50	73	13	30	21	9	.808
≥50	85	20	26	24	15	
Sex						
Male	85	15	31	28	11	.582
Female	73	18	25	17	13	
Complaint (months)						
<12	83	20	29	20	14	.504
≥12	75	13	27	25	10	
Site						
Minor salivary gland	108	21	31	38	18	.026
Major salivary gland	50	12	25	7	6	
Tumour diameter (cm)						
≤1	24	5	6	10	3	.825
1 ~ 2	45	11	18	5	11	
≥2	89	17	32	30	10	
Clinical stage						
Ⅰ+Ⅱ	70	26	17	18	9	.007
Ⅲ+Ⅳ	88	7	39	27	15	
Histological subtype						
Cribriform	72	19	27	22	4	.005
Tubular	49	9	19	15	6	
Solid	37	5	10	8	14	
Involvement of surgical margin						
Affect	48	8	11	16	13	.006
Free	110	25	45	29	11	
Local regional recurrence						
Positive	41	5	10	15	11	.002
Negative	117	28	46	30	13	
Distant metastasis						
Positive	46	7	13	15	11	.027
Negative	112	26	43	30	13	

Then, we ulteriorly analysed the relationship between CXCR4/CXCL12 level and PNI in human SACC tissues. The positive rate of CXCR4 in non‐PNI group (non‐PNI), far from nerve group (PNI‐far) as well as nerve invasion frontier group (PNI‐frontier), was 64.2% (52/81), 84.4% (65/77) and 94.8% (73/77) in SACC specimens, respectively. The distinction of the CXCR4 expression level between non‐PNI and PNI‐far, PNI‐far and PNI‐frontier was significant in statistics (*P* = .001, *P* = .003) (Figure 1B). There was no difference in CXCL12 expression between non‐PNI and PNI‐far, PNI‐far and PNI‐frontier in SACC (*P* > .05). These suggested that high CXCR4 expression might contribute to the presence of PNI, while CXCL12 had not direct association with PNI in SACC.

Kaplan‐Meier curves further revealed that SACC patients with high level of CXCR4 expression had a longer overall survival (OS) time than those with low level or without CXCR4 expression (Figure 1C). Multivariate analysis for prognosis revealed that CXCR4 expression, clinical staging, local relapse, PNI and distance metastases were constructive and meaningful prognostic factors (*P* < .001).

In addition, CXCL12 was highly expressed in microglia BV2 and dorsal root nerve, while CXCR4 was dramatically expressed in SACC cell lines (SACC‐83 and SACC‐LM) and SACC tissue. Although SACC‐LM presented a higher level of CXCR4 than SACC‐83, there was no statistic difference (*P* > .05, Figure 1D).

### 3.2 CXCL12 improved the levels of EMT and Schwann cell‐associated hallmarks of SACC cells

**FIGURE 2:**
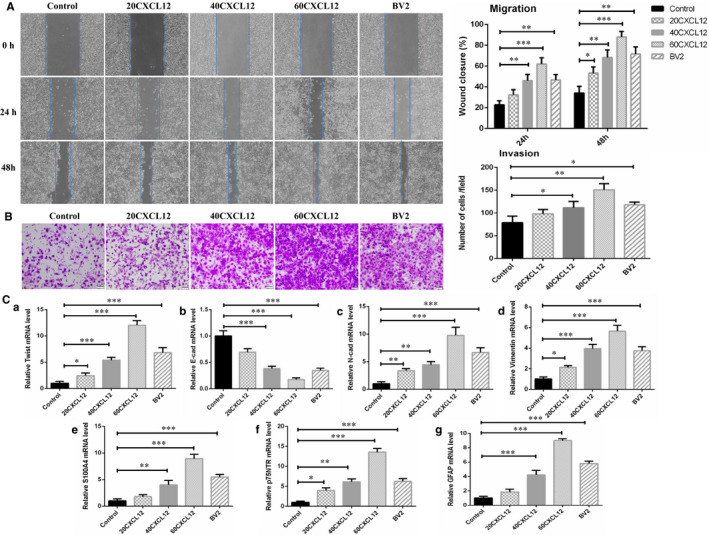
CXCL12 improved the levels of EMT and Schwann cell‐associated hallmarks of SACC‐LM cells. A, SACC‐LM migration was investigated via scratch assay stimulated by different concentrations of cytokine CXCL12 (20 ng/mL, 40 ng/mL, 60 ng/mL) or supernatant of microglia BV2 cells, (40×). B, SACC‐LM invasion was studied using transwell invasion assay stimulated by different concentrations of cytokine CXCL12 (20 ng/ml, 40 ng/ml, 60 ng/ml) or supernatant of microglia BV2 cells, (100×). C, qRT‐PCR analyses assessed the influence of CXCL12/CXCR4 axis on the mRNA level of EMT relevant genes (Twist, E‐cad, N‐cad and Vimentin) together with Schwann cell hallmarks (S100A4, p75NTR and GFAP) in SACC‐LM cells. *P < .05, **P < .01, ***P < .001

It has been known that CXCL12/CXCR4 axis plays a pivotal role in the neurotropism of cancer cells.^23^, ^26^ Hence, we liked to explore the reason why high CXCR4 expression associated with the presence of PNI, but CXCL12 did not present direct relationship with PNI in SACC cases. SACC‐LM cells were stimulated by different concentrations of CXCL12 (20 ng/mL, 40 ng/mL, 60 ng/mL) or supernatant of microglia BV2 cells. The results showed that compared to control group, migratory and invasive capability of SACC‐LM cells were upgraded along with elevated expression level of EMT‐associated genes (Twist, N‐cad, Vimentin and E‐cad) and Schwann cell hallmarks (S100A4, p75NTR and GFAP) in a concentration‐dependent manner by qRT‐PCR (Figure 2A‐C). The same data were found in SACC‐83 cells. These results indicated that CXCL12 might involve in regulating the differentiation of tumour cells into Schwann‐like cells.

### 3.3 CXCL12 promoted migratory, invasive and PNI capacity of SACC cells through CXCR4

**FIGURE 3:**
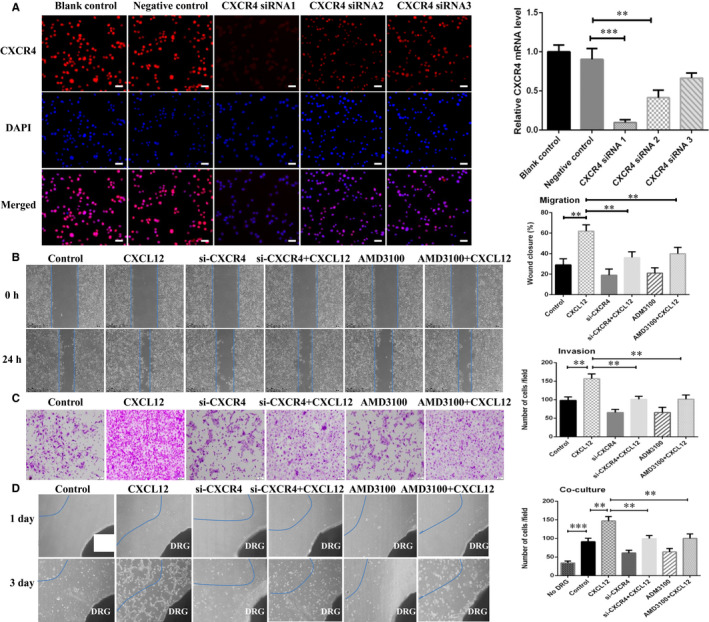
CXCL12 promoted migratory, invasive and PNI capacity of SACC cells through CXCR4. A, Immunofluorescence imaging and qRT‐PCR analysis for transfection efficiency of CXCR4 knockdown by siRNA in SACC‐LM cells, (200×). B, CXCR4 knockdown by siRNA or blockade by AMD3100 inhibited migration of SACC‐LM cells, (40×). C, CXCR4 knockdown by siRNA or blockade by AMD3100 weakened the invasiveness of SACC‐LM cells, (100×). D, CXCR4 knockdown by siRNA or blockade by AMD3100 impaired the transfer competence of SACC‐LM cells towards DRG. DRG indicates dorsal root ganglia (40×). **P < .01, ***P < .001

Subsequently, CXCR4 expression in SACC cells was inhibited by siRNA treatment (Figure 3A). The results confirmed that compared with control group, siRNA‐induced CXCR4 knockdown significantly impaired the migration (Figure 3B), invasion (Figure 3C) and PNI ability (Figure 3D), induced a cytoskeletal collapse and reduced pseudo foot formation by TRITC‐phalloidin staining, down‐regulated the expressive level of EMT associate genes and Schwann cell hallmarks of SACC‐LM cells using qRT‐PCR analysis after stimulated by CXCL12 (60 ng/mL) or supernatant of microglia BV2 cells. The same effect was observed in SACC cells with AMD3100‐mediated CXCR4 blockade. This indicated that CXCL12/CXCR4 was responsible for migratory, invasive and PNI ability of SACC cells.

### 3.4 Twist/S100A4 axis was required in CXCL12/CXCR4 promoting EMT and PNI of SACC cells

**FIGURE 4:**
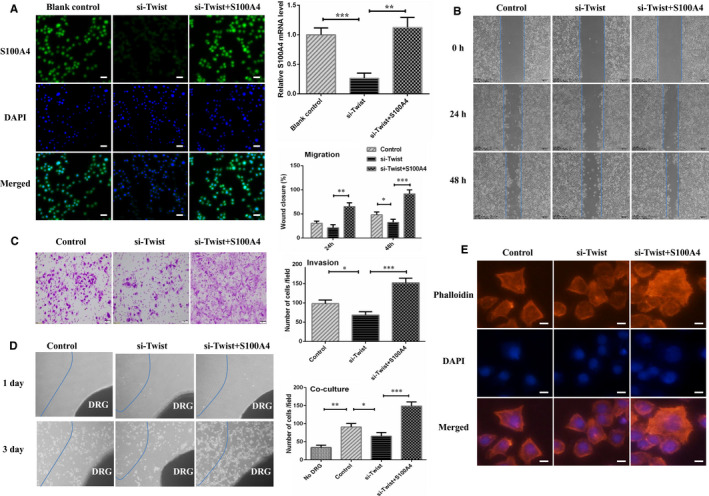
Overexpression of S100A4 promoted SACC cell migratory, invasive and PNI. A, Immunofluorescence and RT‐PCR results displayed that S100A4 level was increased in S100A4 plasmid group of SACC‐LM cells, (200×). B, S100A4 overexpressing SACC‐LM cells emerged advanced transfer capacity. C, S100A4 overexpressing SACC‐LM cells showed contributory invasive ability. D, S100A4 overexpression accelerated SACC‐LM cells transfer towards the DRG in vitro co‐culture assay, (40×). E, TRITC‐phalloidin staining showed that S100A4 overexpression in SACC‐LM stimulated skeleton rearrangement and more pseudo foot formation, (200×). *P < .05, **P < .01, ***P < .001

Then, to further investigate the effect of S100A4 and Twist induced EMT in CXCL12/CXCR4 regulating PNI of SACC, we used siRNA to silence Twist after the sensitization of CXCL12/CXCR4 axis in SACC cells. Silencing Twist reduced migratory, invasive and PNI ability, and down‐regulated EMT‐associated genes and Schwann cell hallmarks mediated by sensitization of CXCL12/CXCR4 axis in SACC cells. Then, we overexpressed S100A4 in SACC‐LM cells with S100A4 overexpressing plasmid, and the transfection efficiency was certificated by immunofluorescence and qRT‐PCR (Figure 4A). The data showed that SACC‐LM cells overexpressing S100A4 rescued the migration, invasion and PNI ability and displayed fibroblast‐like phenotype and pseudo foot, frequently (Figure 4B‐E). This indicated that CXCL12/CXCR4 promoted SACC cell migration, invasion and PNI partly through Twist/S100A4 axis.

### 3.5 CXCL12/CXCR4‐mediated EMT promoted PNI in SACC xenograft model

**FIGURE 5:**
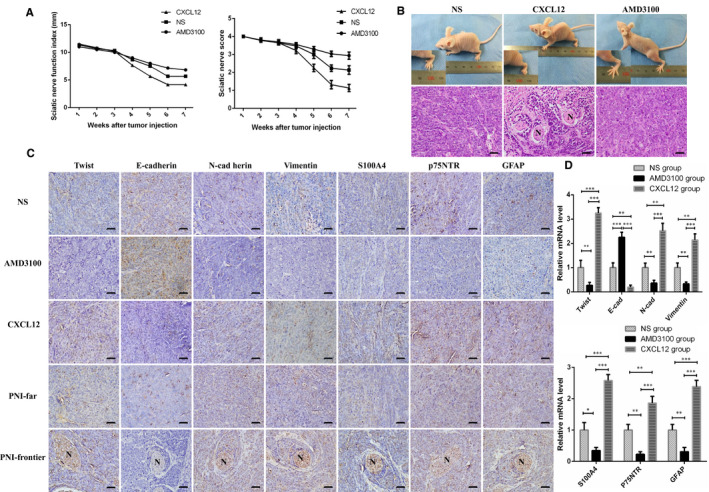
CXCL12/CXCR4‐mediated EMT promoted PNI in SACC xenograft model. A, Sciatic nerve function index and function of hind limb was assessed. B, Function of hind limb of the mouse was observed, and the results of HE staining was displayed in SACC xenograft model, (200×). C, Immunohistochemical staining showed that the level of EMT‐related proteins and Schwann cell hallmarks in CXCL12 group were remarkably higher relative to saline group, and the protein level in saline group was higher than AMD3100 group, and the expression at PNI‐frontier was higher than PNI‐far group, (200×). D, qRT‐PCR analysis exhibited that mRNA level of EMT‐related genes and Schwann cell hallmarks in CXCL12 group were significantly higher than that in saline group, and the mRNA level in saline group was significantly higher than AMD3100 group. *P < .05, **P < .01, ***P < .001

To confirm these findings in vitro, PNI model with nude mice was established. The results showed that sciatic nerve function index of hind limb was prominently decreased in CXCL12 group compared with saline group and AMD3100 group at the 5th week, and saline group significantly decreased than AMD3100 group at the 6th week (Figure 5A). Functional assessment demonstrated that the mouse in the CXCL12 group began to undergo hind limb dysfunction at the 4th week compared with AMD3100 group. At the 5th week, hind limb function in CXCL12 group was dramatically worse than saline group, and saline group was worse than AMD3100 group (Figure 5A). Xenografted tumours were excised for HE staining at 7th week, and the results manifested that the presence of PNI in CXCL12 group, saline group, AMD3100 group was 85.7% (6/7), 16.7% (1/6) and 0 (0/7), respectively. The incidence of PNI in CXCL12‐treated group was remarkably higher than AMD3100 group and saline group (*P* < .05, Figure 5B). The data of immunohistochemical staining (Figure 5C) and qRT‐PCR (Figure 5D) showed that the level of EMT‐related genes as well as Schwann cell hallmarks in CXCL12 group was notably higher than saline group, and the level in saline group was strikingly higher than that in AMD3100 group. In addition, the expression level of EMT‐related proteins and Schwann cell hallmarks in PNI‐frontier were higher than PNI‐far group (Figure 5C). This indicated that CXCL12/CXCR4 signalling might involve in the course of EMT and Schwann‐like differentiation in PNI of SACC.

### 3.6 CXCR4 expression closely connected with the level of EMT‐related markers in SACC specimens

**FIGURE 6:**
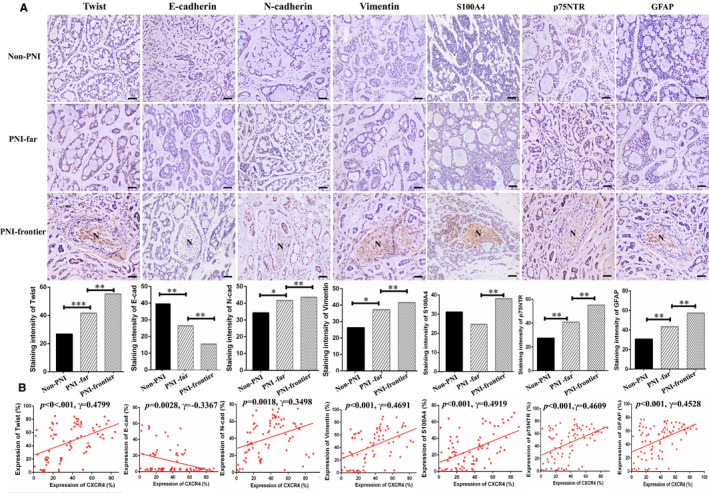
CXCR4 expression closely connected with the level of EMT‐related markers in SACC specimens. A, Immunostaining of EMT hallmarks (Twist, E‐cad, N‐cad and Vimentin) as well as Schwann cells relevant genes (S100A4, p75NTR and GFAP) in different tissue. ‘Normal gland’ indicates normal salivary gland. ‘Non‐PNI’ indicates SACC without PNI. ‘PNI‐far’ indicates SACC far away nerve site. ‘PNI‐frontier’ indicates nerve invasion frontier, (200×). B, Correlation of CXCR4 level with EMT together with Schwann cell‐related hallmarks at nerve invasion front in SACC patients. CXCR4 level was closely relative to Twist, Vimentin, S100A4, p75NTR and GFAP but notably inversely interrelated with the E‐cad level

CXCR4 pathway has been certificated to influence EMT of cancer cells in vitro.^8^, ^9^, ^27^ Immunostaining of EMT‐associated proteins (N‐cad, Vimentin, Twist, E‐cad) as well as Schwann cell hallmarks (S100A4, p75NTR, GFAP) was conducted in 158 SACC and 20 normal glands specimens (Figure 6A). The correlative analysis between the CXCR4 expression and the level of EMT‐associated proteins and Schwann cell hallmarks at PNI‐frontier was carried out. As shown in the illustration, the level of CXCR4 was notably inverse interrelated with the E‐cad (γ = −0.3367, *P* = .0028), while positive related to the level of Twist (γ = 0.4799, *P* ˂ 0.001), N‐cad (γ = 0.3498, *P* = .0018), Vimentin (γ = 0.4691, *P* < .001), S100A4 (γ = 0.4919, *P* < .001), p75NTR (γ = 0.4609, *P* < .001) and GFAP (γ = 0.4528, *P* < .001). This indicated that CXCL12/CXCR4 signalling might promote EMT to induce Schwann‐like differentiation in SACC (Figure 6B).

## 4 DISCUSSION

The occurrence of PNI in SACC not just leads to symptoms of neurological impairment, including facioplegia, soreness and numbness, but it even is detrimental to disease‐free survival. Accordingly, the mechanisms responsible for PNI of SACC should be fully elucidated. In this thesis, we identified that CXCR4 expression was prominently correlated with tumour location, clinical staging, histological subtype, implication of resection margin, tumour relapse and metastasis. This was in line with previous findings that CXCR4 was positively associated with cancer‐related deaths.^28^, ^29^ Besides, the results presented here also demonstrated that CXCR4 in nerve invasion frontier was strikingly expressed. CXCL12 was abundantly expressed by nerve cells. This phenomenon was also observed in the specimens of pancreatic cancer with PNI.^26^ This revealed that the intertwining between CXCL12, mainly expressed by nerve tissue in the SACC specimens with PNI and CXCR4, strongly expressed by tumour cells at nerve invasion frontier might offer favourable terms for the initiation of PNI in SACC.

To verify the key role of CXCL12/CXCR4 in promotion of PNI in SACC, we extended our investigations in vitro. The results showed that cytokine CXCL12 or supernatant of microglia BV2 cells expressing CXCL12 induced enhanced migratory, invasive and PNI ability of SACC cells in a dose‐dependent fashion, which could be reserved after silencing CXCR4 with siRNA or blocking CXCR4 of SACC cells with AMD3100, which was verified to function as antagonist of CXCR4, thus inhibiting intracranial merisis of primary cerebral tumour.^30^ This confirmed that CXCL12/CXCR4 contributed to the PNI of SACC cells.

To encroach on nerve, reduced intercellular adhesion and enhanced migration and invasion capability were required in cancer cells.^31^ EMT, a biological process, by which polarized epithelial cells converted into mesenchymal phenotype cells and acquired a motile mesenchymal cytoskeleton, was a pivotal procedure in migration and invasion of malignant tumours.^32^, ^33^ In this study, we identified that the level of E‐cad was reduced and Twist, N‐cad and Vimentin were elevated at nerve invasion front than the site far away from nerve. The correlation analysis suggested that CXCR4 level was inversely correlated with protein level of E‐cad, whereas strongly positively correlated with Twist, N‐cad and Vimentin expression at nerve invasion front. This was in accordance with the biology characterization and facility of CXCR4, which was essential to steer invasive and metastatic potency through EMT. For example, Guo et al noted that CXCL12/CXCR4 could accelerate invasion of glioblastoma through enhancing the EMT.^34^ Raschioni et al reported that activated CXCR4/CXCL12 pathway sustained the aggressiveness of breast cancer through initiating EMT.^35^ These suggested that CXCR4 might participate in PNI of SACC through mediating EMT.

Twist was proved to be a crucial orchestrator inducing EMT in tumour progression.^36^ To determine the role of Twist in the neurophilic invasion of SACC cells, Twist in SACC cells were knockdown using siRNA. As expected, the results demonstrated that silencing Twist inhibited cancer cell migratory, invasive and PNI ability, accompanied by reduced EMT‐associated genes and Schwann cell hallmarks level. Further, we noticed that the migratory, invasive and PNI ability of SACC cells was significantly enhanced, and fibroblast‐like phenotype and pseudo foot was captured frequently after S100A4 overexpression by plasmid. These findings suggested that CXCL12/CXCR4 might provoke PNI by regulating the differentiation of tumour cells into Schwann‐like cells through Twist/S100A4 in SACC.

Accumulating evidences have indicated that tumour cells differentiating towards Schwann‐like cell could accelerate the predisposition for PNI of tumours.^19^, ^37^, ^38^, ^39^ In this study, the level of S100A4, p75NTR and GFAP, which had been characterized as Schwann cell markers,^11^, ^12^ was evidently increased at nerve invasion frontier. And the relevancy analysis indicated that CXCR4 expression level was positively correlated with S100A4, p75NTR and GFAP level at nerve invasion front. These revealed that tumour cell differentiating towards Schwann‐like cell might occur and CXCR4 might encourage transversion of Schwann‐like cell differentiation in PNI of SACC.

All in all, the present study demonstrated that CXCL12 was mainly expressed by nerve tissue and CXCR4 was highly expressed by SACC cells at nerve invasion front in specimens with PNI. The activation of CXCL12/CXCR4 might accelerate PNI through enhancing the initiation of EMT to induce tumour cells develop into Schwann‐like cells, triggering cytoskeletal rearrangement, thus acquiring a promotional migrated and invasive ability in SACC.

## CONFLICT OF INTEREST

The authors declare that they have no conflicts of interest.

## AUTHOR CONTRIBUTION

**Mei Zhang:** Data curation (equal); Formal analysis (equal); Investigation (equal); Methodology (equal); Visualization (equal); Writing‐original draft (equal). **Min Zheng:** Investigation (equal); Methodology (equal); Visualization (equal). **Li Dai:** Data curation (equal); Formal analysis (equal). **Wei‐long Zhang:** Data curation (equal); Formal analysis (equal). **Hua‐yang Fan:** Data curation (equal); Formal analysis (equal). **Xiang‐hua Yu:** Data curation (equal); Formal analysis (equal). **Xin Pang:** Data curation (equal); Formal analysis (equal). **Peng Liao:** Data curation (equal); Formal analysis (equal). **Bing‐jun Chen:** Data curation (equal); Formal analysis (equal). **Sha‐sha Wang:** Data curation (equal); Formal analysis (equal). **Mingxin Cao:** Data curation (equal); Formal analysis (equal). **Xiang‐rui Ma:** Writing‐review & editing (equal). **Ya‐ling Tang:** Conceptualization (equal); Funding acquisition (equal); Writing‐review & editing (equal). **Xinhua Liang:** Conceptualization (equal); Funding acquisition (equal); Writing‐review & editing (equal).

## ETHICS APPROVAL AND INFORMED CONSENT

The research on human tissue specimens was in accordance with ethical regulations of the Institutional Ethics Committee of the West China Medical Center, Sichuan University, China (WCHSIRB‐D‐2016‐207, WCHSIRB‐D‐2017‐120). Written informed consent was obtained from the participants. All studies involving animal were conducted complied with the guidelines of the Subcommittee on Research and Animal Care (SRAC) of Sichuan University (WCHSIRB‐D‐2016‐191).

## CONSENT FOR PUBLICATION

Not applicable.

## Supporting information

Supplementary Material

## Data Availability

All data generated or analysed during this study are included in this published article [and its supplementary information files].

## References

[1] Liebig C, Ayala G, Wilks JA, Berger DH, Albo D. Perineural invasion in cancer: a review of the literature. Cancer. 2009;115:3379‐3391. 19484787 10.1002/cncr.24396

[2] Gupta A, Veness M, De'Ambrosis B, Selva D, Huilgol SC. Management of squamous cell and basal cell carcinomas of the head and neck with perineural invasion. Aust J Dermatol. 2016;57:3‐13. 10.1111/ajd.12314 25759949

[3] Pour PM, Bell RH, Batra SK. Neural invasion in the staging of pancreatic cancer. Pancreas. 2003;26:322‐325. 12717262 10.1097/00006676-200305000-00002

[4] Feng FY, Qian Y, Stenmark MH, et al. Perineural invasion predicts increased recurrence, metastasis, and death from prostate cancer following treatment with dose‐escalated radiation therapy. Int J Radiat Oncol Biol Phys. 2011;81:e361‐e367. 21820250 10.1016/j.ijrobp.2011.04.048

[5] Liebig C, Ayala G, Wilks J, et al. Perineural invasion is an independent predictor of outcome in colorectal cancer. J Clin Oncol. 2009;27:5131‐5137. 19738119 10.1200/JCO.2009.22.4949 PMC2773472

[6] Conley‐LaComb MK, Semaan L, Singareddy R, et al. Pharmacological targeting of CXCL12/CXCR4 signaling in prostate cancer bone metastasis. Mol Cancer. 2016;15:68. 27809841 10.1186/s12943-016-0552-0 PMC5093938

[7] Luo T, Liu H, Feng W, et al. Adipocytes enhance expression of osteoclast adhesion‐related molecules through the CXCL12/CXCR4 signalling pathway. Cell Prolif. 2017;50(3):e12317. 10.1111/cpr.12317 PMC6529116 27868262

[8] Yang F, Takagaki Y, Yoshitomi Y. Inhibition of dipeptidyl peptidase‐4 accelerates epithelial‐mesenchymal transition and breast cancer metastasis via the CXCL12/CXCR4/mTOR axis. Can Res. 2019;79:735‐746. 10.1158/0008-5472.CAN-18-0620 30584072

[9] Cheng Y, Song Y, Qu J, et al. The chemokine receptor CXCR4 and c‐MET cooperatively promote epithelial‐mesenchymal transition in gastric cancer cells. Transl Oncol. 2018;11:487‐497. 29494948 10.1016/j.tranon.2018.02.002 PMC5884220

[10] Richardson PJ. CXCR4 and glioblastoma. Anticancer Agents Med Chem. 2016;16:59‐74. 26299663 10.2174/1871520615666150824153032

[11] Ziegler L, Grigoryan S, Yang IH, Thakor NV, Goldstein RS. Efficient generation of Schwann cells from human embryonic stem cell‐derived neurospheres. Stem Cell Rev Rep. 2011;7:394‐403. 21052870 10.1007/s12015-010-9198-2

[12] Ramli K, Aminath Gasim I, Ahmad AA, et al. Human bone marrow‐derived MSCs spontaneously express specific Schwann cell markers. Cell Biol Int. 2019;43:233‐252. 30362196 10.1002/cbin.11067

[13] Carlson JA, Dickersin GR, Sober AJ, Barnhill RL. Desmoplastic neurotropic melanoma. A clinicopathologic analysis of 28 cases. Cancer. 1995;75:478‐494. 7812919 10.1002/1097-0142(19950115)75:2<478::aid-cncr2820750211>3.0.co;2-o

[14] Kwon SY, Bae YK, Gu MJ, et al. Neuroendocrine differentiation correlates with hormone receptor expression and decreased survival in patients with invasive breast carcinoma. Histopathology. 2014;64:647‐659. 24117859 10.1111/his.12306

[15] Park K, Chen Z, MacDonald TY, et al. Prostate cancer with Paneth cell‐like neuroendocrine differentiation has recognizable histomorphology and harbors AURKA gene amplification. Hum Pathol. 2014;45:2136‐2143. 25128228 10.1016/j.humpath.2014.06.008 PMC4414025

[16] Lee SJ, Choi SY, Kim WJ, et al. Combined aberrant expression of E‐cadherin and S100A4, but not β‐catenin is associated with disease‐free survival and overall survival in colorectal cancer patients. Diagn Pathol. 2013;8:99. 23783026 10.1186/1746-1596-8-99 PMC3728147

[17] Demir IE, Boldis A, Pfitzinger PL, et al. Investigation of Schwann cells at neoplastic cell sites before the onset of cancer invasion. J Natl Cancer Inst. 2014;106:dju184. 25106646 10.1093/jnci/dju184

[18] Deborde S, Wong RJ. How Schwann cells facilitate cancer progression in nerves. Cell Mol Life Sci. 2017;74:4405‐4420. 28631007 10.1007/s00018-017-2578-x PMC5665723

[19] Shan C, Wei J, Hou R, et al. Schwann cells promote EMT and the Schwann‐like differentiation of salivary adenoid cystic carcinoma cells via the BDNF/TrkB axis. Oncol Rep. 2016;35:427‐435. 26530352 10.3892/or.2015.4366

[20] Ayala GE, Wheeler TM, Shine HD, et al. In vitro dorsal root ganglia and human prostate cell line interaction: redefining perineural invasion in prostate cancer. Prostate. 2001;49:213‐223. 11746267 10.1002/pros.1137

[21] Ceyhan GO, Demir IE, Altintas B, et al. Neural invasion in pancreatic cancer: a mutual tropism between neurons and cancer cells. Biochem Biophys Res Comm. 2008;374:442‐447. 18640096 10.1016/j.bbrc.2008.07.035

[22] Li X, Wang Z, Ma Q, et al. Sonic hedgehog paracrine signaling activates stromal cells to promote perineural invasion in pancreatic cancer. Clin Cancer Res. 2014;20:4326‐4338. 24947933 10.1158/1078-0432.CCR-13-3426

[23] Takaoka K, Hidaka S, Hashitani S, et al. Effect of a nitric oxide synthase inhibitor and a CXC chemokine receptor‐4 antagonist on tumor growth and metastasis in a xenotransplanted mouse model of adenoid cystic carcinoma of the oral floor. Int J Oncol. 2013;43:737‐745. 23835861 10.3892/ijo.2013.2011

[24] Gil Z, Rein A, Brader P, et al. Nerve‐sparing therapy with oncolytic herpes virus for cancers with neural invasion. Clin Cancer Res. 2007;13:6479‐6485. 17975160 10.1158/1078-0432.CCR-07-1639

[25] Gil Z, Kelly KJ, Brader P, Shah JP, Fong Y, Wong RJ. Utility of a herpes oncolytic virus for the detection of neural invasion by cancer. Neoplasia (New York, NY). 2008;10:347‐353. 10.1593/neo.07981 PMC2288543 18392138

[26] Xu Q, Wang Z, Chen X, et al. Stromal‐derived factor‐1α/CXCL12‐CXCR4 chemotactic pathway promotes perineural invasion in pancreatic cancer. Oncotarget. 2015;6:4717‐4732. 25605248 10.18632/oncotarget.3069 PMC4467110

[27] Jäger B, Klatt D, Plappert L, et al. CXCR4/MIF axis amplifies tumor growth and epithelial‐mesenchymal interaction in non‐small cell lung cancer. Cell Signal. 2020;73:109672. 32428553 10.1016/j.cellsig.2020.109672

[28] Zhang J, Chen J, Wo D, et al. LRP6 ectodomain prevents SDF‐1/CXCR4‐induced breast cancer metastasis to lung. Clin Cancer Res. 2019;25:4832‐4845. 31010839 10.1158/1078-0432.CCR-18-3557

[29] Chen IX, Chauhan VP, Posada J, et al. Blocking CXCR4 alleviates desmoplasia, increases T‐lymphocyte infiltration, and improves immunotherapy in metastatic breast cancer. Proc Natl Acad Sci USA. 2019;116:4558‐4566. 30700545 10.1073/pnas.1815515116 PMC6410779

[30] Rubin JB, Kung AL, Klein RS, et al. A small‐molecule antagonist of CXCR4 inhibits intracranial growth of primary brain tumors. Proc Natl Acad Sci USA. 2003;100:13513‐13518. 14595012 10.1073/pnas.2235846100 PMC263845

[31] Wei C, Yang C, Wang S, et al. Crosstalk between cancer cells and tumor associated macrophages is required for mesenchymal circulating tumor cell‐mediated colorectal cancer metastasis. Mol Cancer. 2019;18:64. 30927925 10.1186/s12943-019-0976-4 PMC6441214

[32] Jiang S, Wang X, Song D, et al. Cholesterol induces epithelial‐to‐mesenchymal transition of prostate cancer cells by suppressing degradation of EGFR through APMAP. Can Res. 2019;79:3063‐3075. 10.1158/0008-5472.CAN-18-3295 30987997

[33] Huergo‐Zapico L, Parodi M, Cantoni C. NK‐cell editing mediates epithelial‐to‐mesenchymal transition via phenotypic and proteomic changes in melanoma cell lines. Can Res. 2018;78:3913‐3925. 10.1158/0008-5472.CAN-17-1891 29752261

[34] Guo X, Xu S, Gao X, et al. Macrophage migration inhibitory factor promotes vasculogenic mimicry formation induced by hypoxia via CXCR4/AKT/EMT pathway in human glioblastoma cells. Oncotarget. 2017;8:80358‐80372. 29113309 10.18632/oncotarget.18673 PMC5655204

[35] Raschioni C, Bottai G, Sagona A, et al. CXCR4/CXCL12 Signaling and protumor macrophages in primary tumors and sentinel lymph nodes are involved in luminal B breast cancer progression. Dis Markers. 2018;2018:5018671. 29849822 10.1155/2018/5018671 PMC5926522

[36] Lai YJ, Yu WN, Kuo SC, et al. CSC‐3436 inhibits TWIST‐induced epithelial‐mesenchymal transition via the suppression of Twist/Bmi1/Akt pathway in head and neck squamous cell carcinoma. J Cell Physiol. 2019;234:9118‐9129. 30341909 10.1002/jcp.27589

[37] Iwamoto S, Odland PB, Piepkorn M, Bothwell M. Evidence that the p75 neurotrophin receptor mediates perineural spread of desmoplastic melanoma. J Am Acad Dermatol. 1996;35:725‐731. 8912568 10.1016/s0190-9622(96)90728-8

[38] Luo XL, Sun MY, Lu CT, Zhou ZH. The role of Schwann cell differentiation in perineural invasion of adenoid cystic and mucoepidermoid carcinoma of the salivary glands. Int J Oral Maxillofac Surg. 2006;35:733‐739. 16513325 10.1016/j.ijom.2006.01.012

[39] Chen W, Dong S, Zhou J, Sun M. Investigation of myoepithelial cell differentiation into Schwann‐like cells in salivary adenoid cystic carcinoma associated with perineural invasion. Mol Med Rep. 2012;6:755‐759. 22842649 10.3892/mmr.2012.1003

