# Supplementary figures and images for: CXCL12/CXCR4 facilitates perineural invasion via induction of the Twist/S100A4 axis in salivary adenoid cystic carcinoma
Source: J Cell Mol Med. 2021 Jun 25;25(16):7901–12. doi: 10.1111/jcmm.16713 (PMC8358865; doi:10.1111/jcmm.16713)

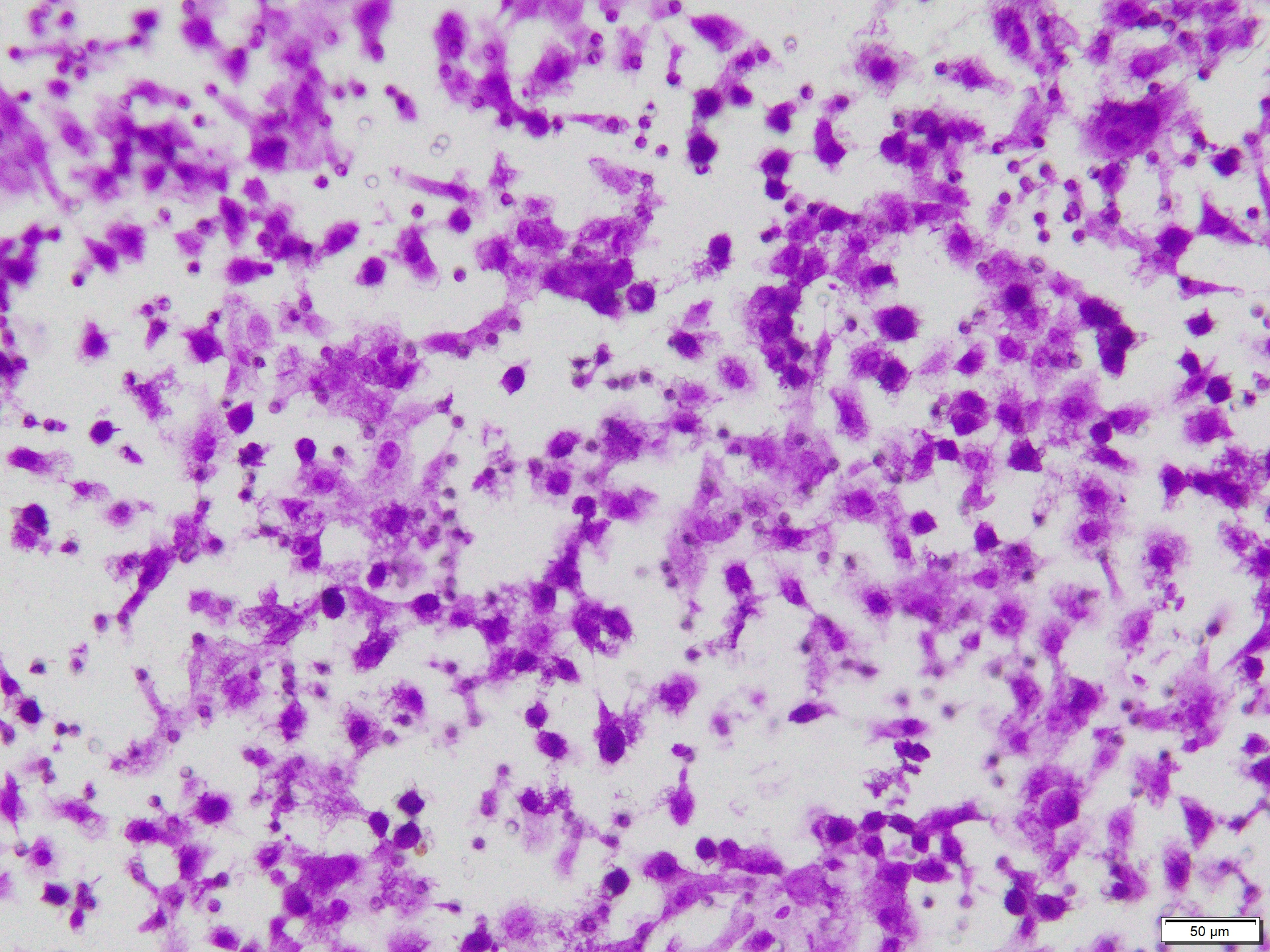

Supplement: Supplementary file 1 — Supplementary Material [file JCMM-25-7901-s001.zip › jcmm16713-sup-0001-Data/Figure 2/Fig 2 Invasion/Fig 2-20CXCL12 invasion.jpg]

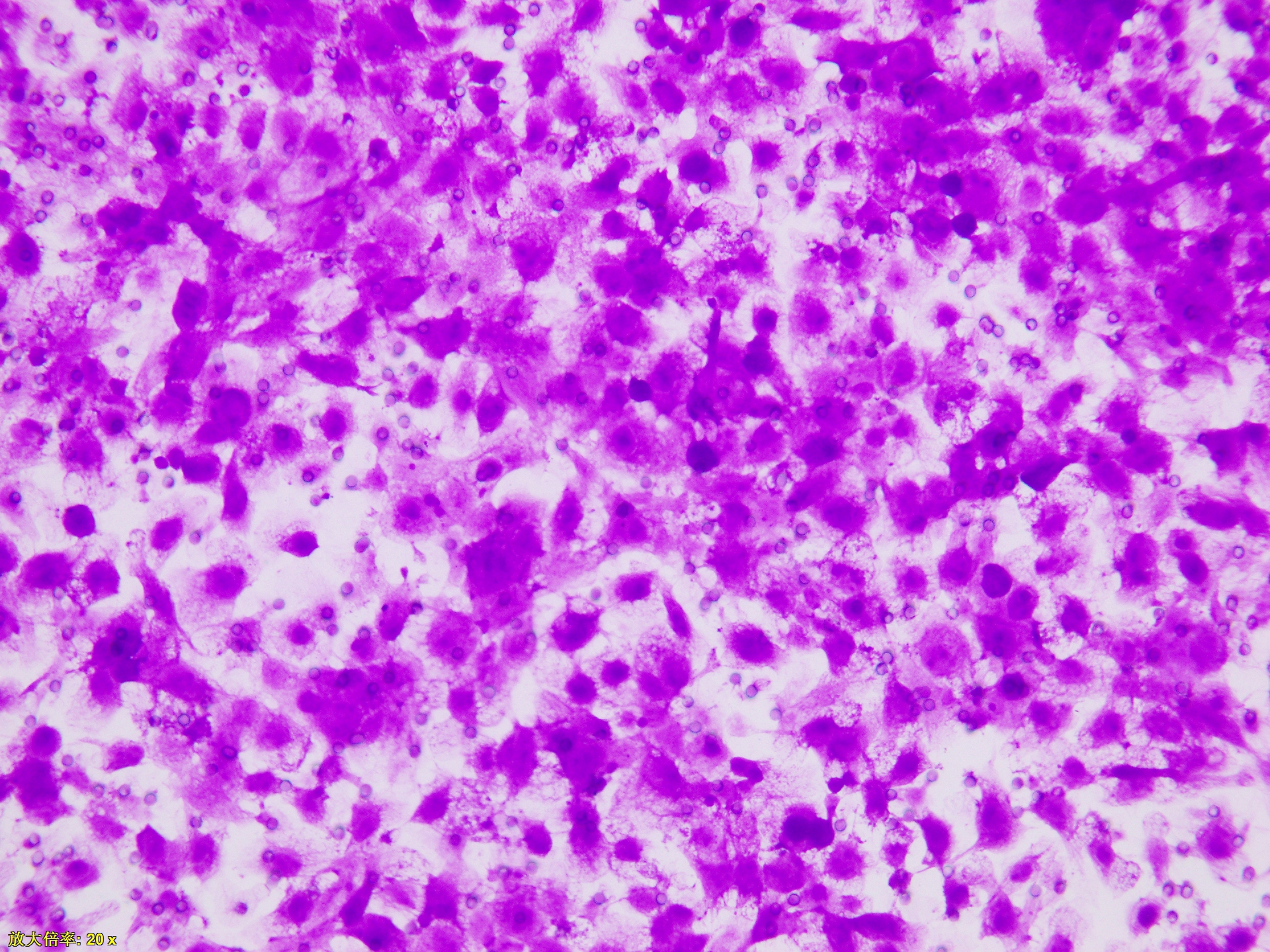

Supplement: Supplementary file 1 — Supplementary Material [file JCMM-25-7901-s001.zip › jcmm16713-sup-0001-Data/Figure 2/Fig 2 Invasion/Fig 2-40CXCL12 invasion.jpg]

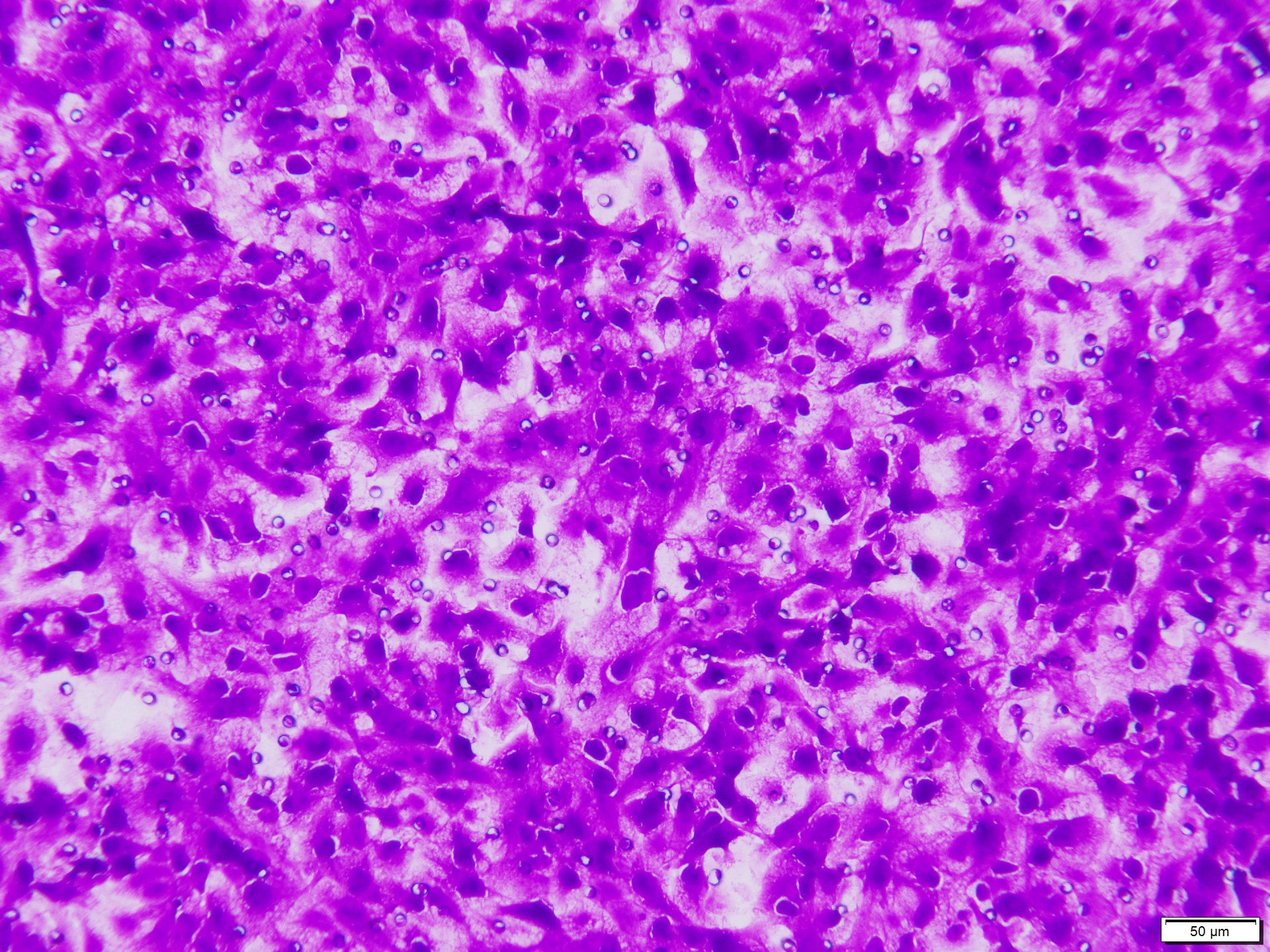

Supplement: Supplementary file 1 — Supplementary Material [file JCMM-25-7901-s001.zip › jcmm16713-sup-0001-Data/Figure 2/Fig 2 Invasion/Fig 2-60CXCL12 invasion.jpg]

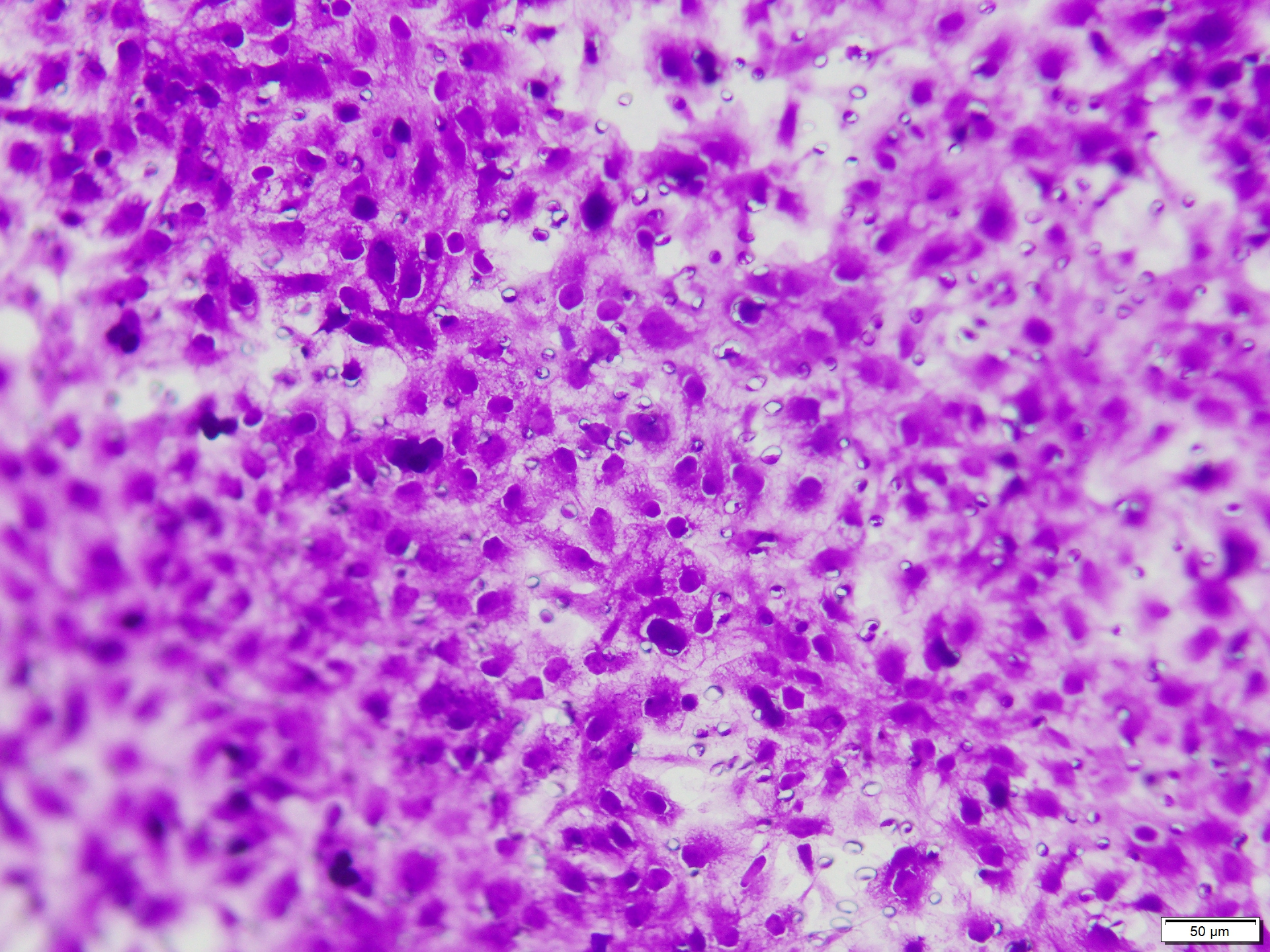

Supplement: Supplementary file 1 — Supplementary Material [file JCMM-25-7901-s001.zip › jcmm16713-sup-0001-Data/Figure 2/Fig 2 Invasion/Fig 2-BV2 invasion.jpg]

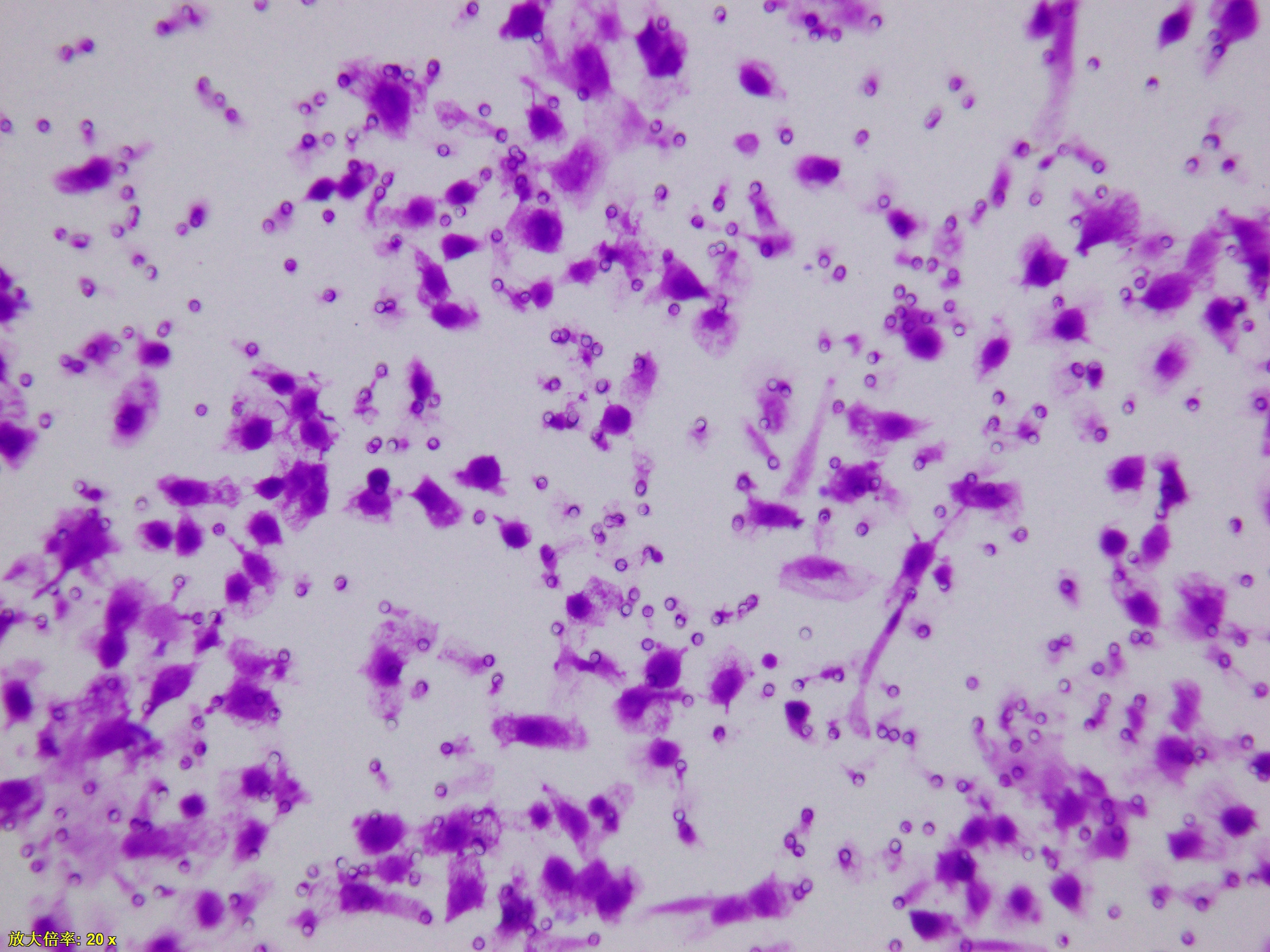

Supplement: Supplementary file 1 — Supplementary Material [file JCMM-25-7901-s001.zip › jcmm16713-sup-0001-Data/Figure 2/Fig 2 Invasion/Fig 2-Control invasion.jpg]

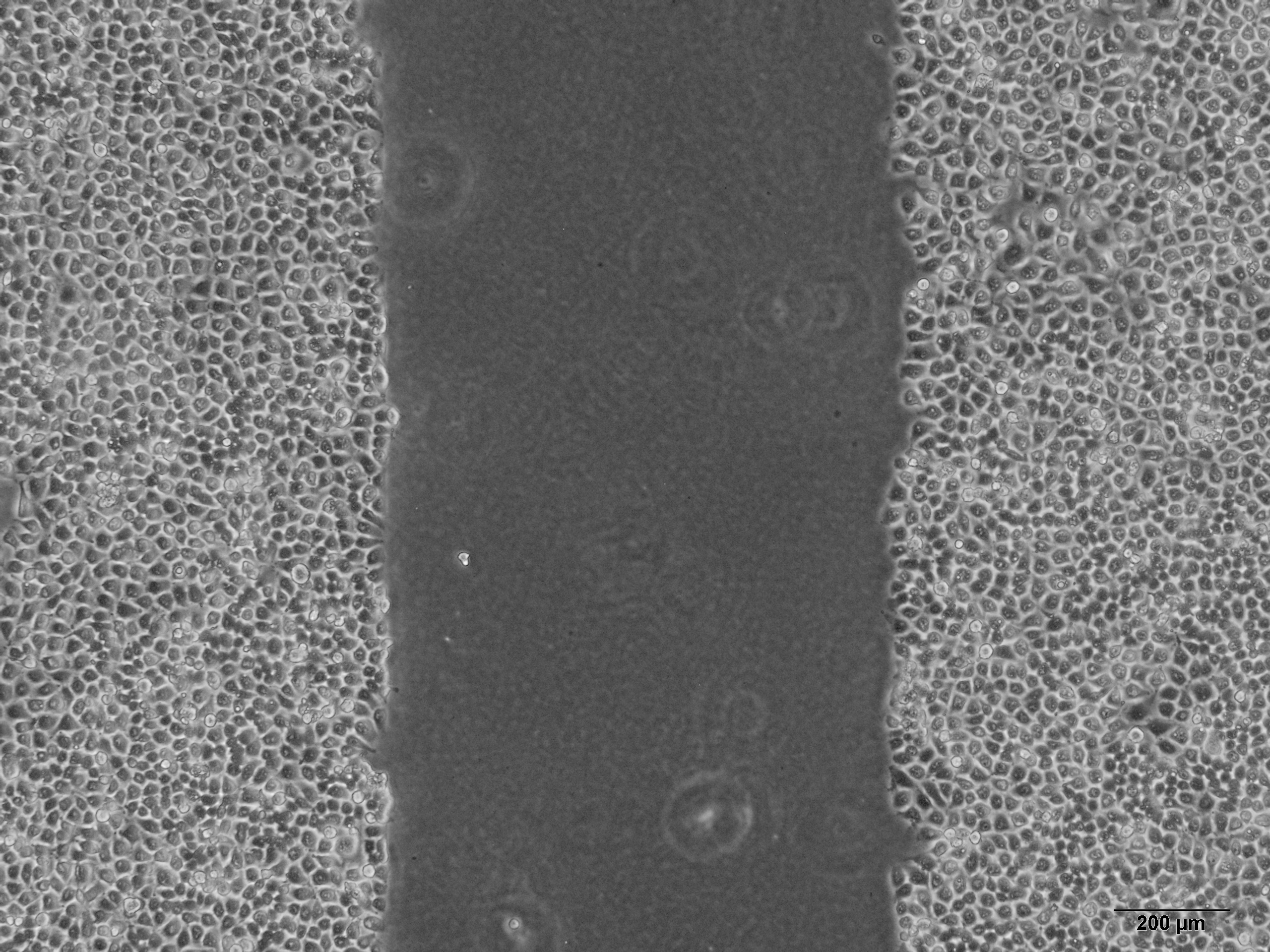

Supplement: Supplementary file 1 — Supplementary Material [file JCMM-25-7901-s001.zip › jcmm16713-sup-0001-Data/Figure 2/Fig 2 Migration/Fig 2-20CXCL12 0h migration.tif]

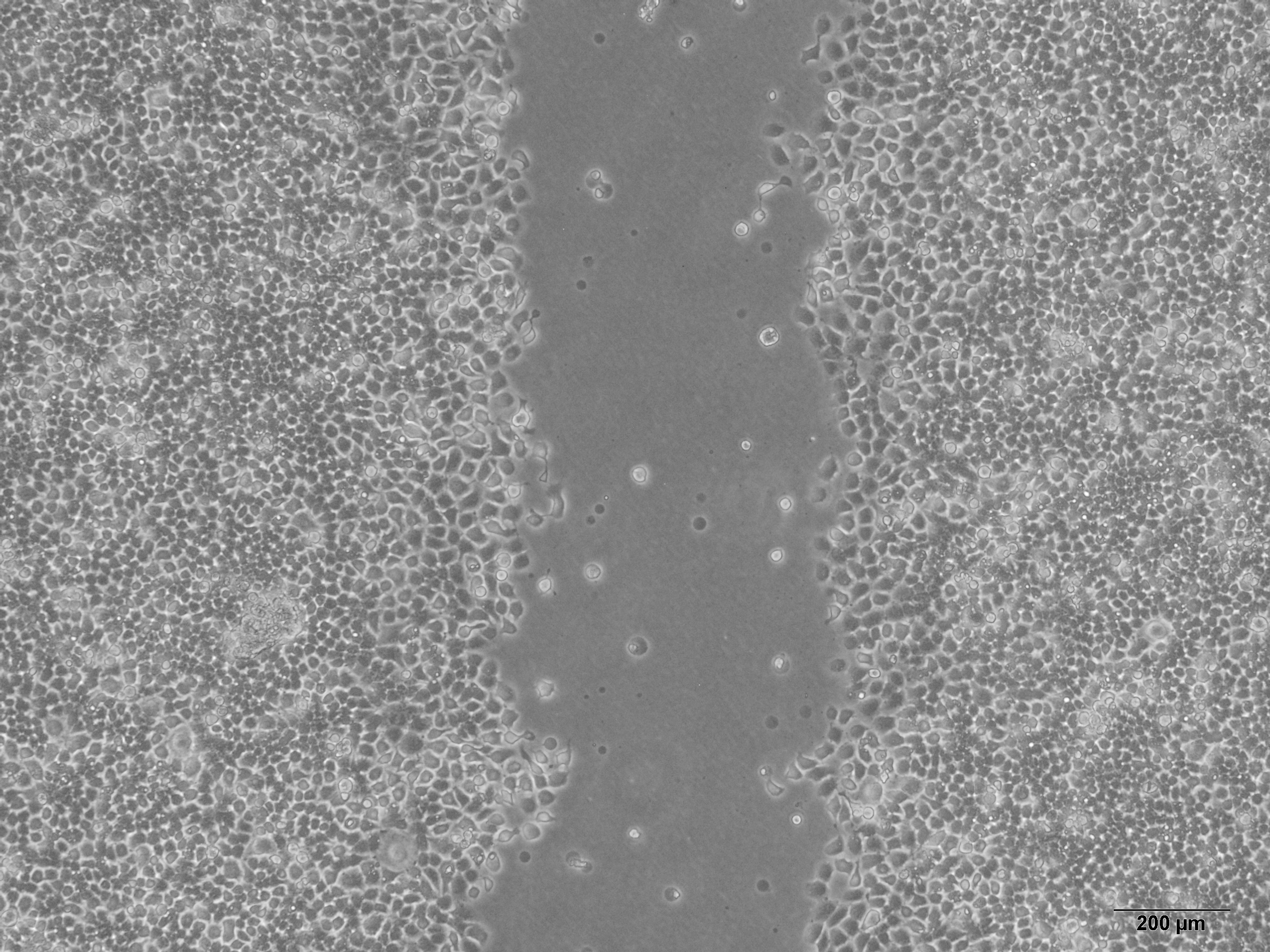

Supplement: Supplementary file 1 — Supplementary Material [file JCMM-25-7901-s001.zip › jcmm16713-sup-0001-Data/Figure 2/Fig 2 Migration/Fig 2-20CXCL12 24h migration.tif]

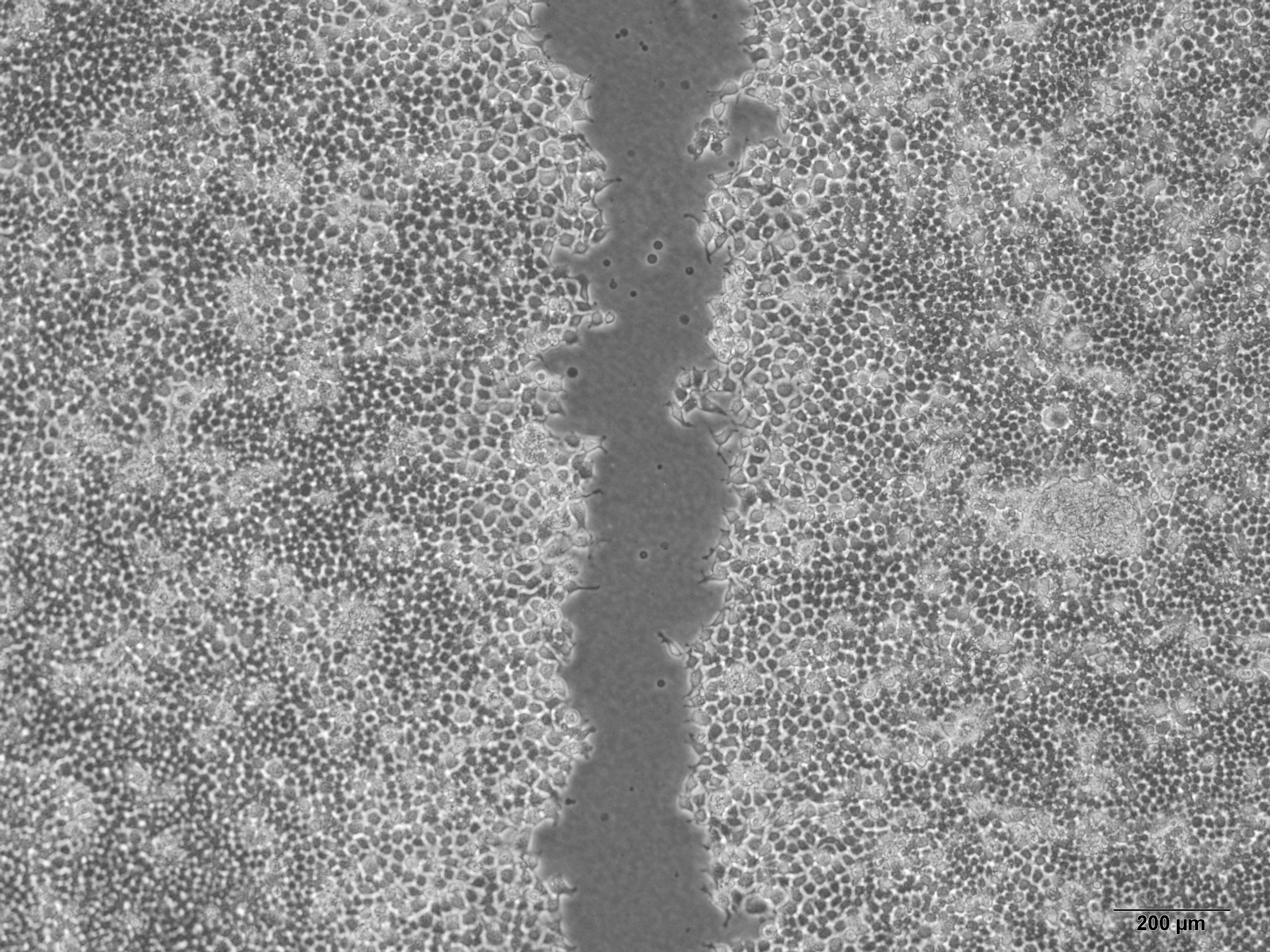

Supplement: Supplementary file 1 — Supplementary Material [file JCMM-25-7901-s001.zip › jcmm16713-sup-0001-Data/Figure 2/Fig 2 Migration/Fig 2-20CXCL12 48h migration.tif]

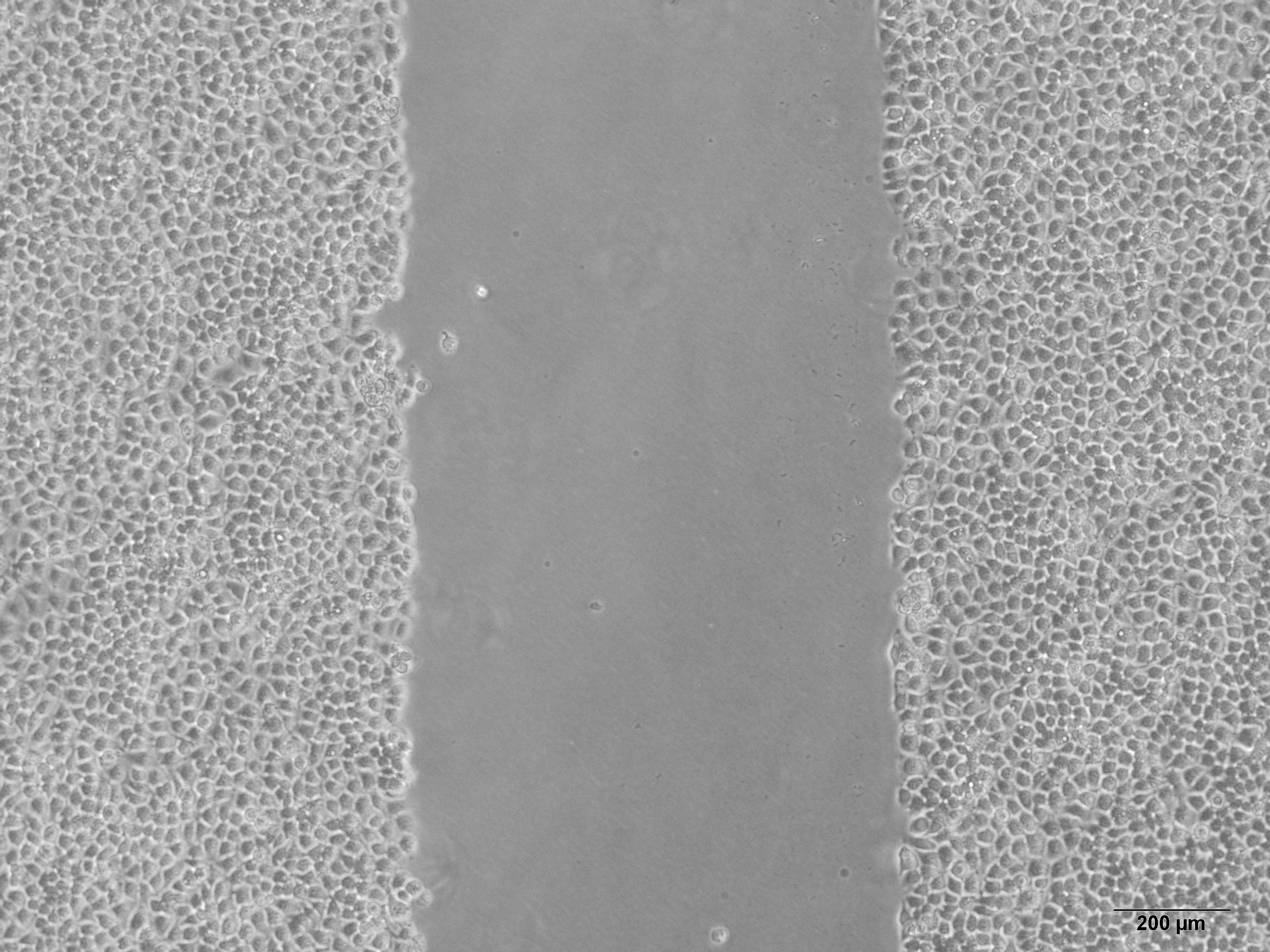

Supplement: Supplementary file 1 — Supplementary Material [file JCMM-25-7901-s001.zip › jcmm16713-sup-0001-Data/Figure 2/Fig 2 Migration/Fig 2-40CXCL12 0h migration.tif]

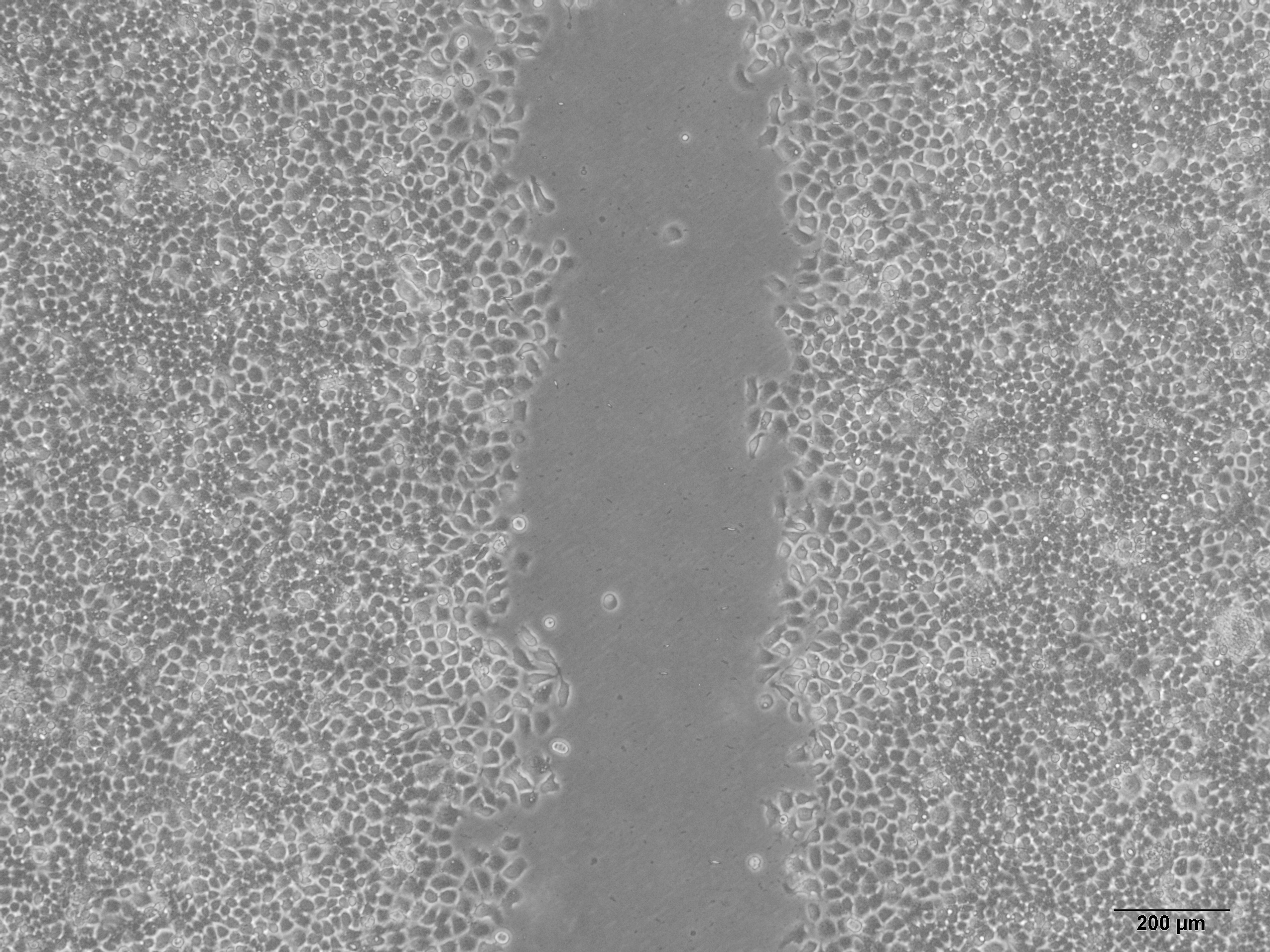

Supplement: Supplementary file 1 — Supplementary Material [file JCMM-25-7901-s001.zip › jcmm16713-sup-0001-Data/Figure 2/Fig 2 Migration/Fig 2-40CXCL12 24h migration.tif]

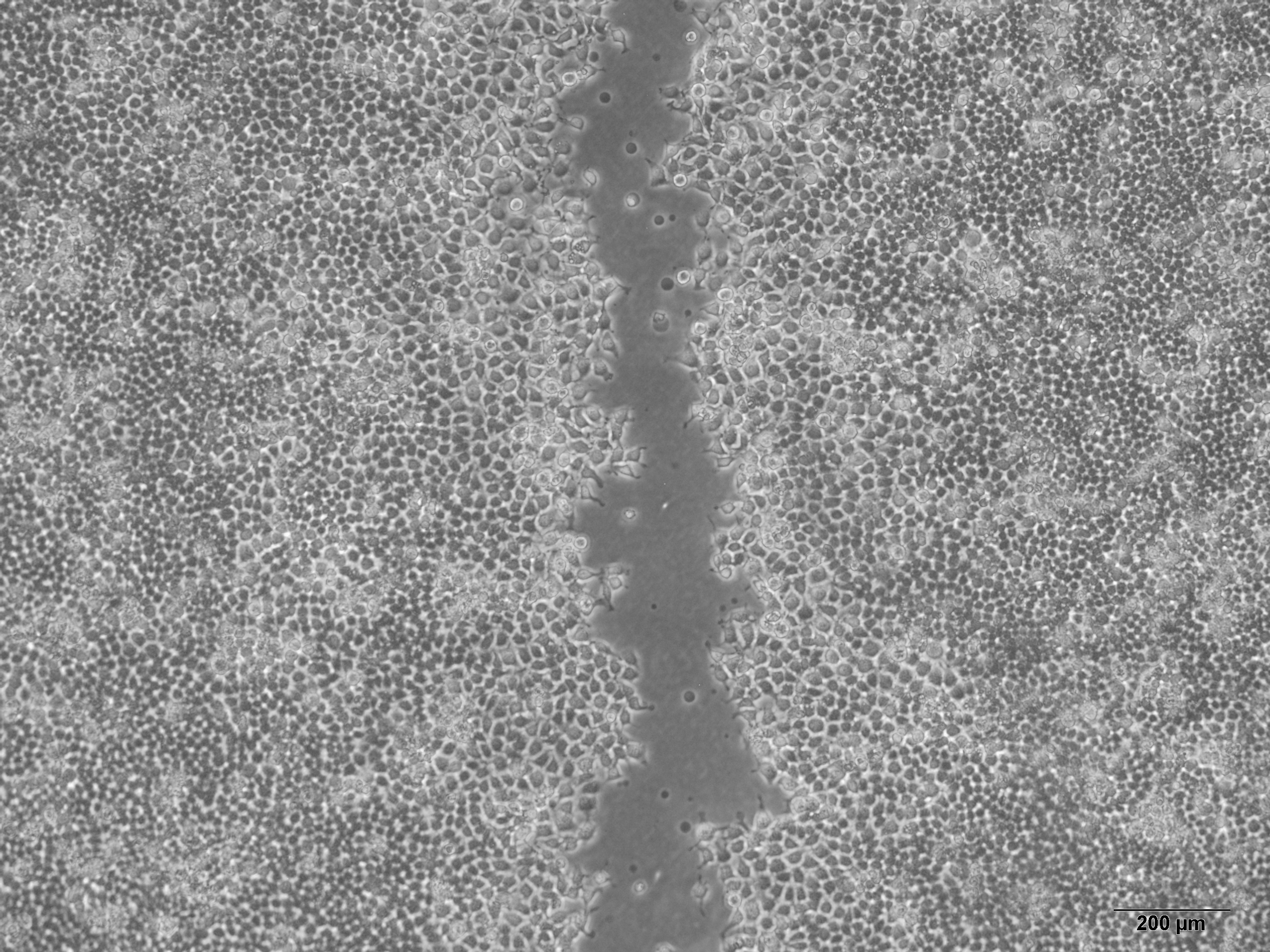

Supplement: Supplementary file 1 — Supplementary Material [file JCMM-25-7901-s001.zip › jcmm16713-sup-0001-Data/Figure 2/Fig 2 Migration/Fig 2-40CXCL12 48h migration.tif]

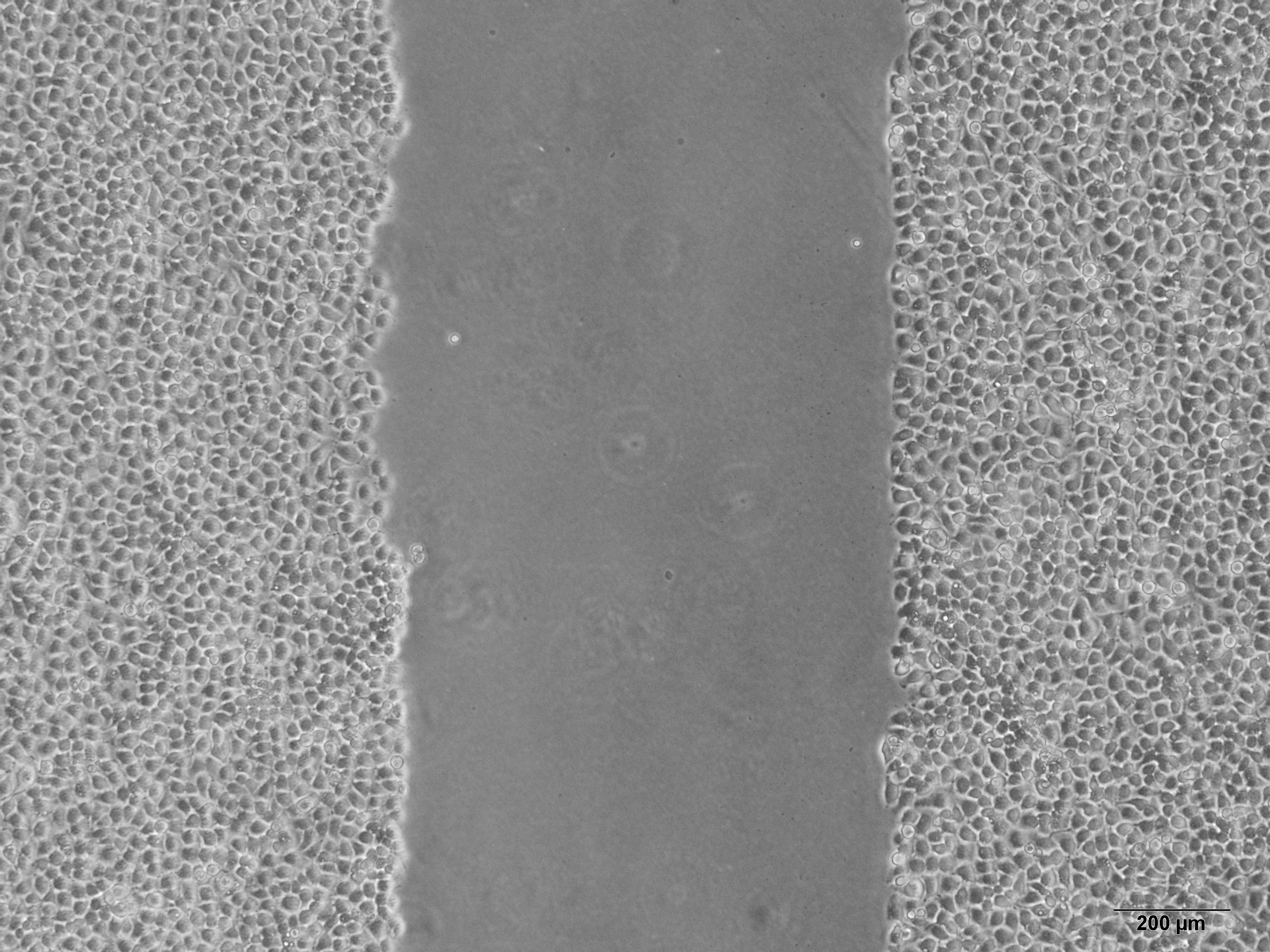

Supplement: Supplementary file 1 — Supplementary Material [file JCMM-25-7901-s001.zip › jcmm16713-sup-0001-Data/Figure 2/Fig 2 Migration/Fig 2-60CXCL12 0h migration.tif]

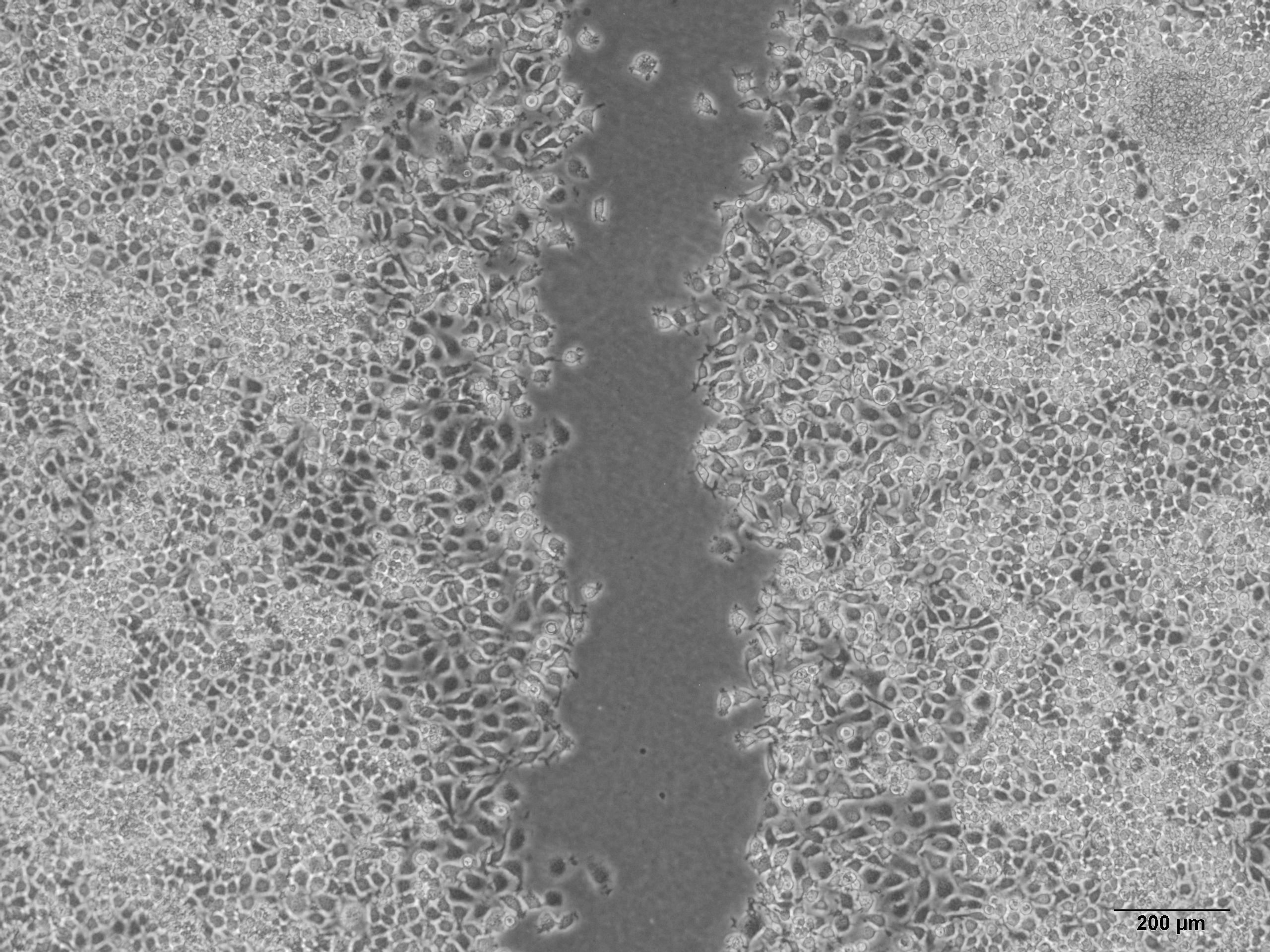

Supplement: Supplementary file 1 — Supplementary Material [file JCMM-25-7901-s001.zip › jcmm16713-sup-0001-Data/Figure 2/Fig 2 Migration/Fig 2-60CXCL12 24h migration.tif]

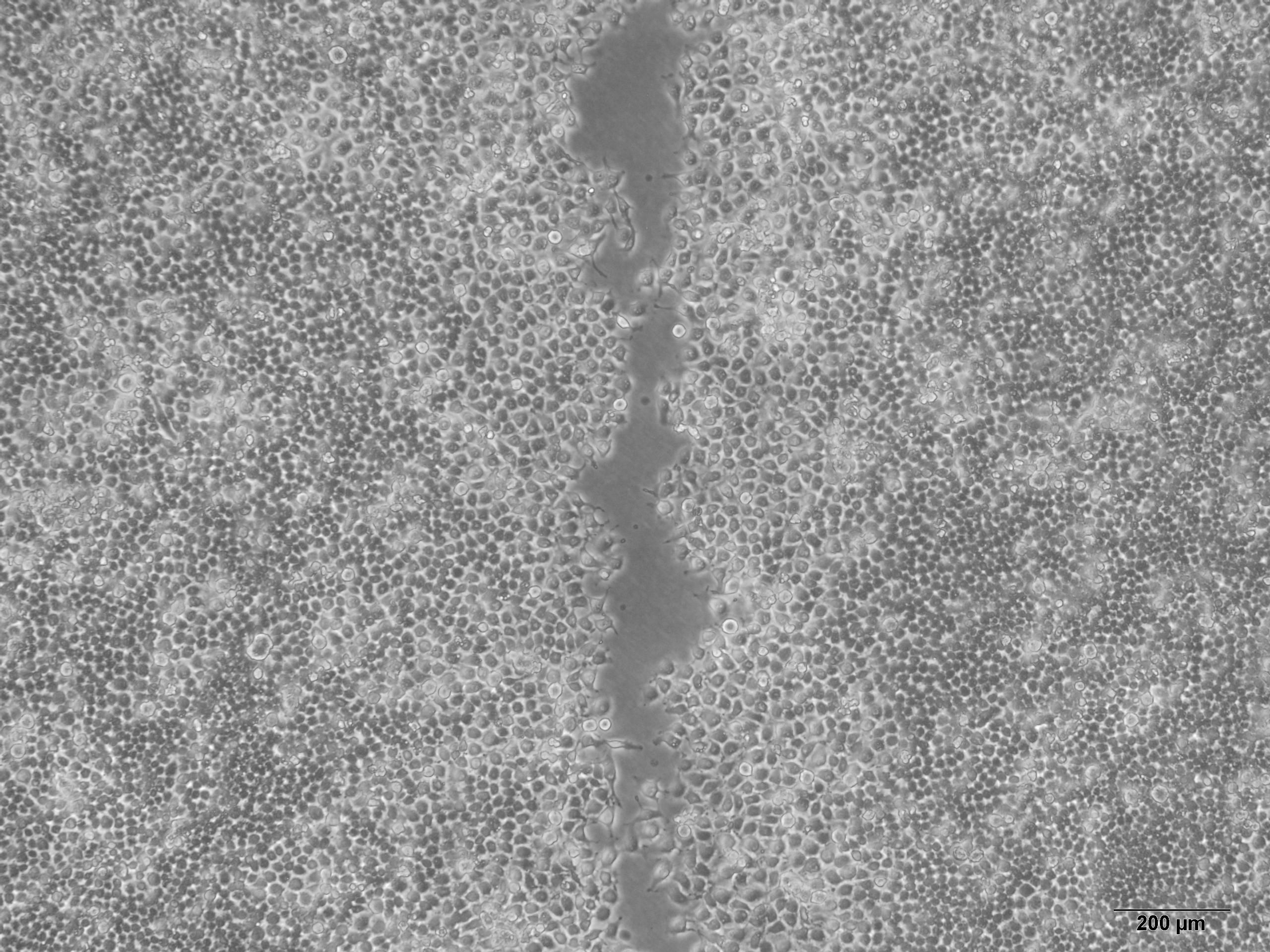

Supplement: Supplementary file 1 — Supplementary Material [file JCMM-25-7901-s001.zip › jcmm16713-sup-0001-Data/Figure 2/Fig 2 Migration/Fig 2-60CXCL12 48h migration.tif]

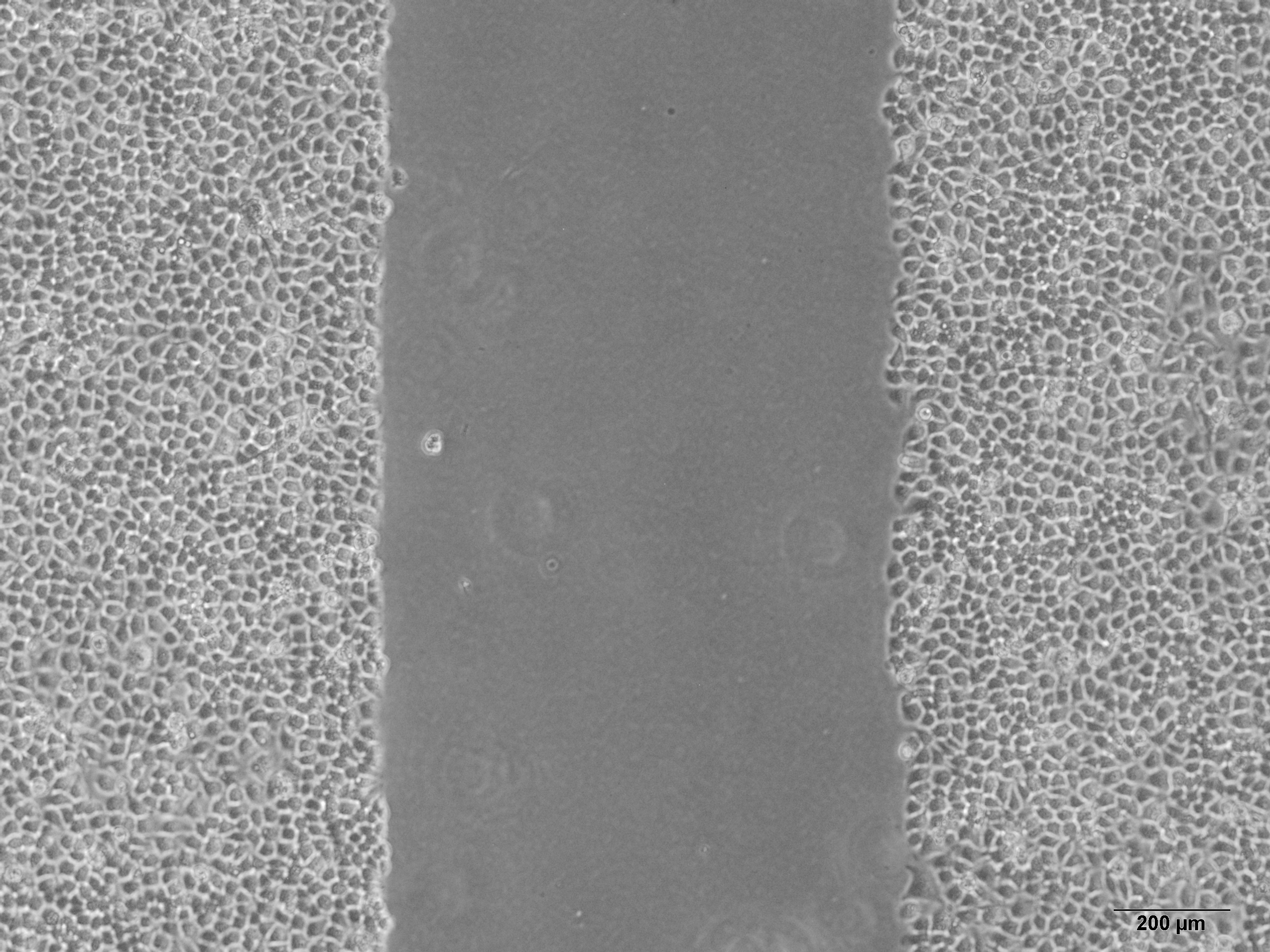

Supplement: Supplementary file 1 — Supplementary Material [file JCMM-25-7901-s001.zip › jcmm16713-sup-0001-Data/Figure 2/Fig 2 Migration/Fig 2-BV2 0h migration.tif]

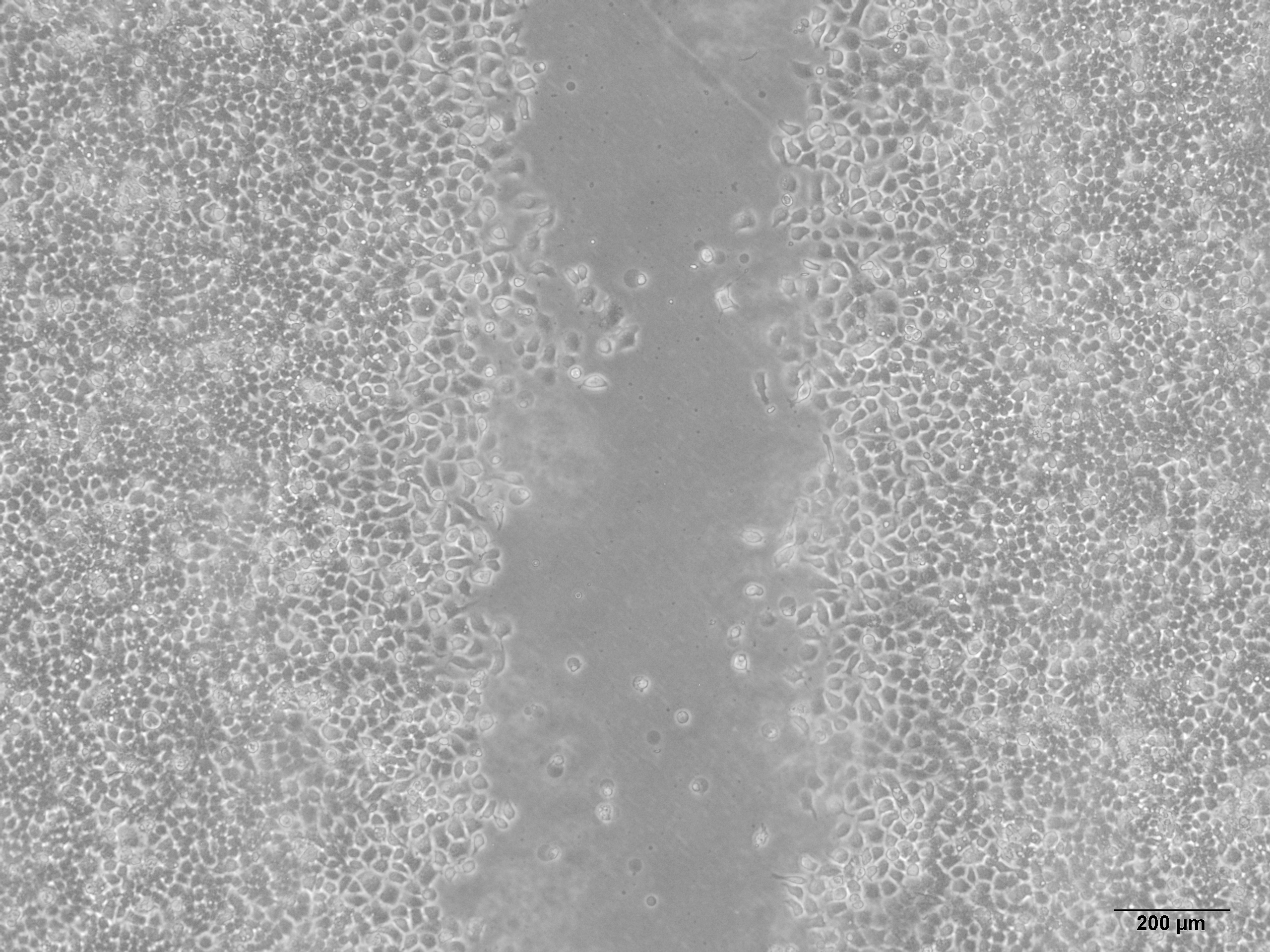

Supplement: Supplementary file 1 — Supplementary Material [file JCMM-25-7901-s001.zip › jcmm16713-sup-0001-Data/Figure 2/Fig 2 Migration/Fig 2-BV2 24h migration.tif]

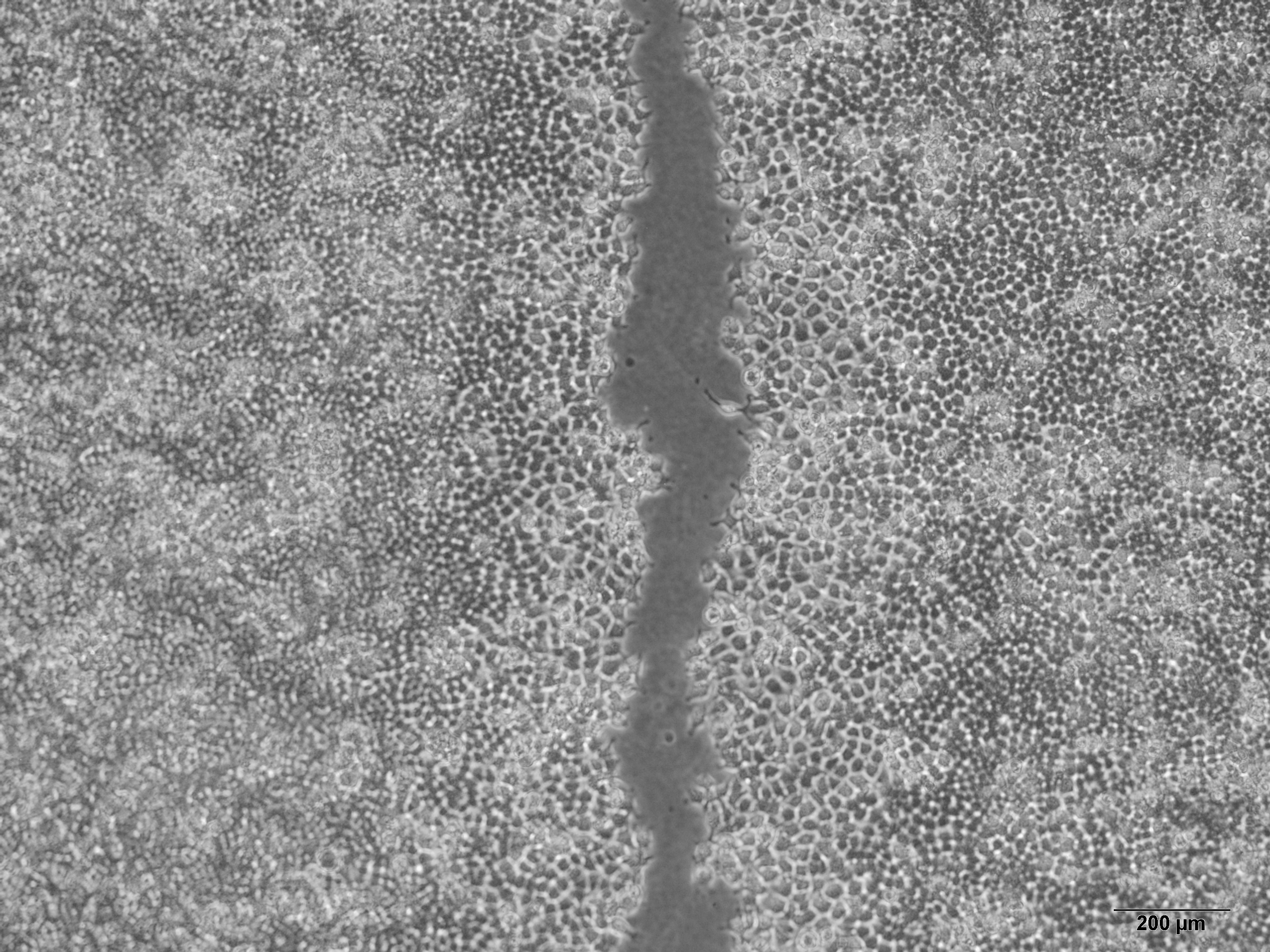

Supplement: Supplementary file 1 — Supplementary Material [file JCMM-25-7901-s001.zip › jcmm16713-sup-0001-Data/Figure 2/Fig 2 Migration/Fig 2-BV2 48h migration.tif]

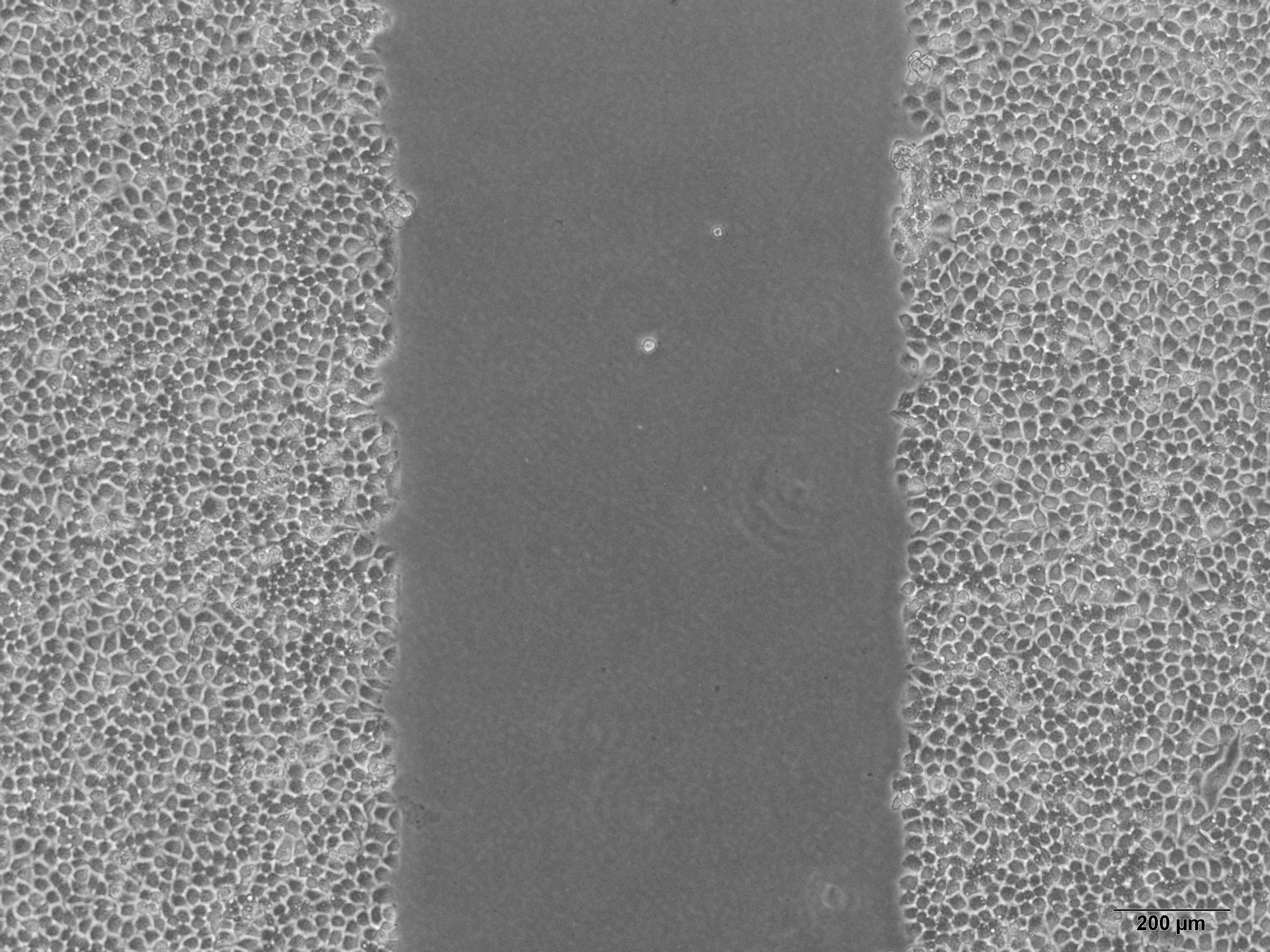

Supplement: Supplementary file 1 — Supplementary Material [file JCMM-25-7901-s001.zip › jcmm16713-sup-0001-Data/Figure 2/Fig 2 Migration/Fig 2-Control 0h migration.tif]

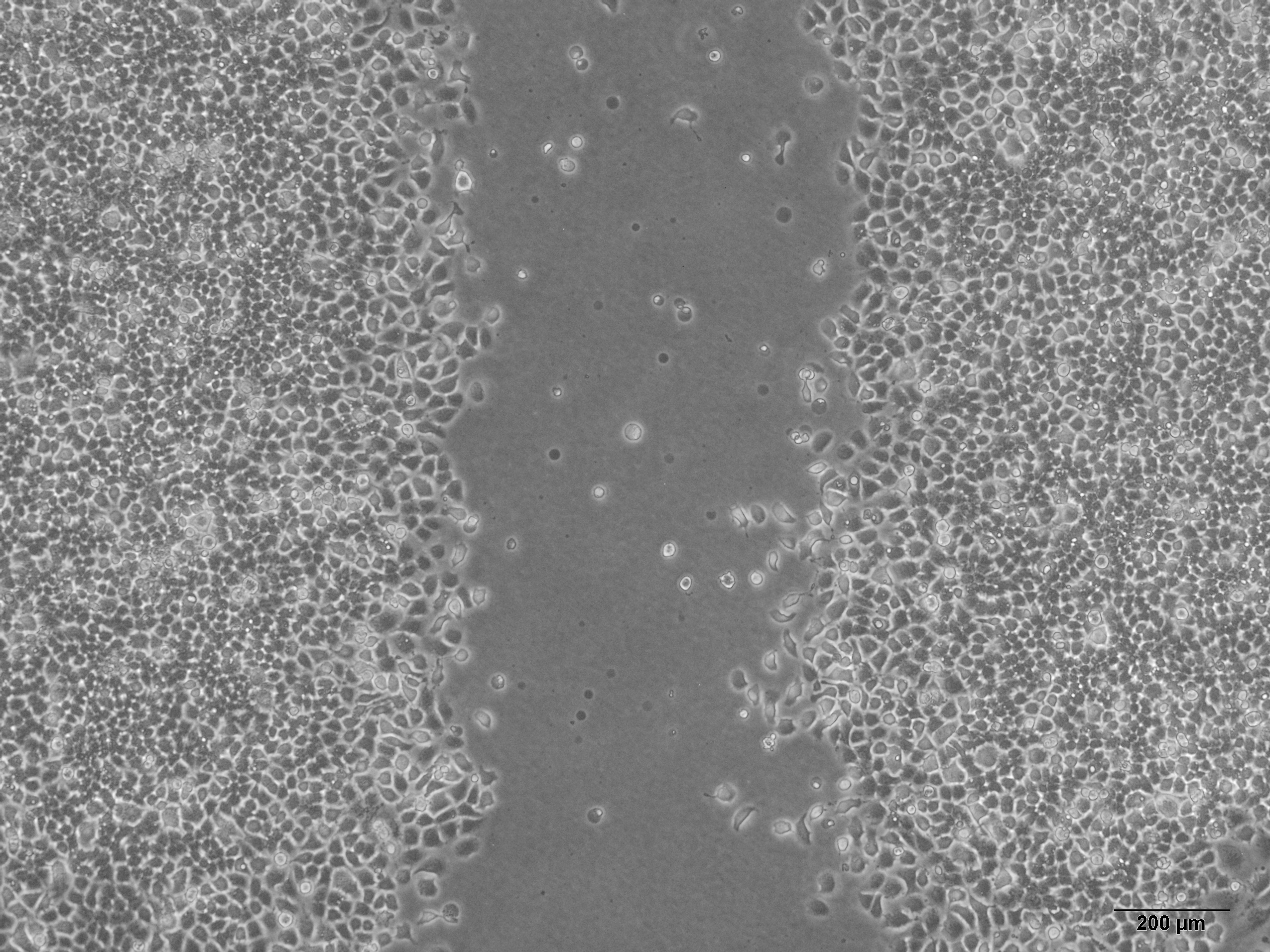

Supplement: Supplementary file 1 — Supplementary Material [file JCMM-25-7901-s001.zip › jcmm16713-sup-0001-Data/Figure 2/Fig 2 Migration/Fig 2-Control 24h migration.tif]

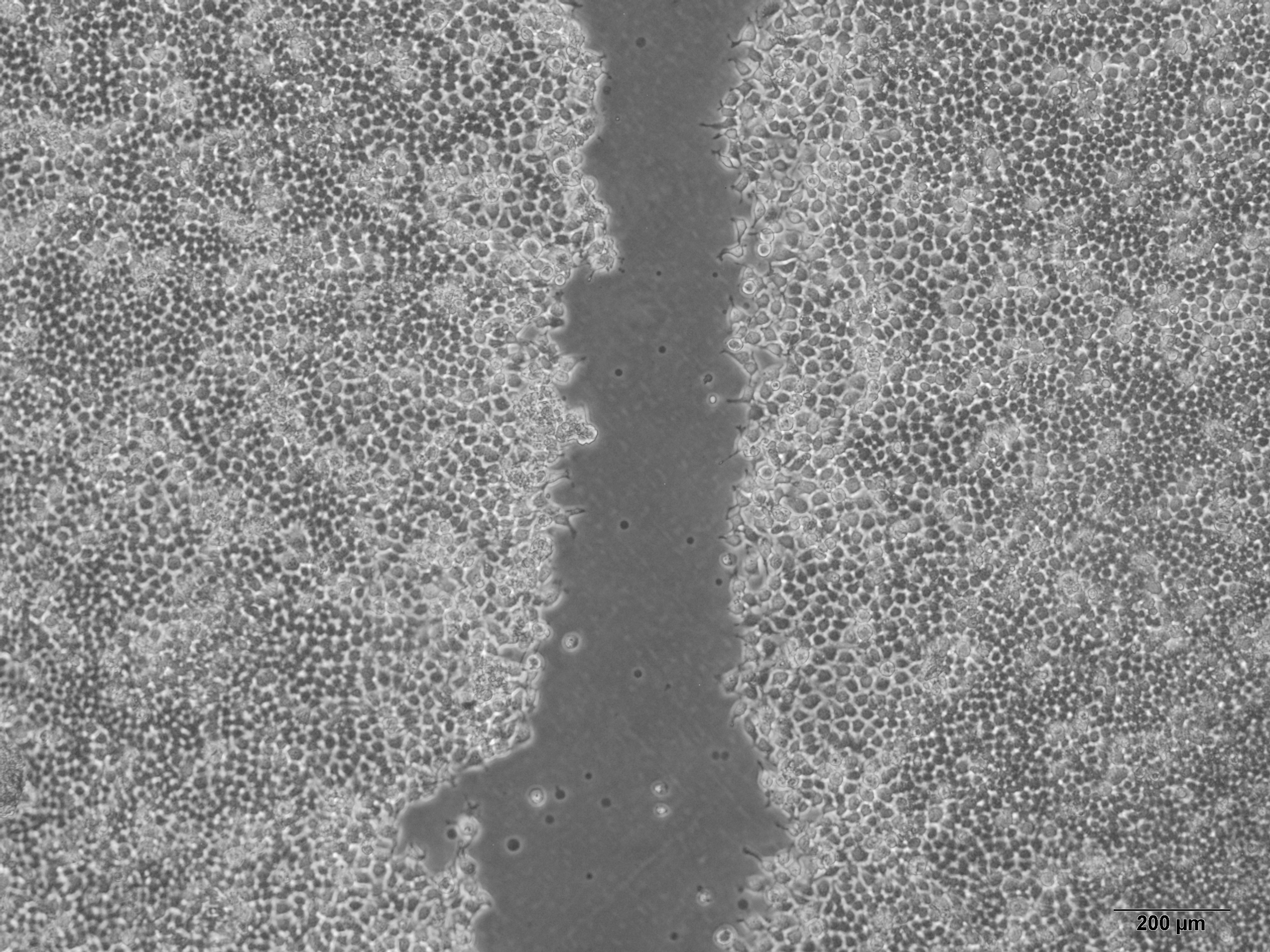

Supplement: Supplementary file 1 — Supplementary Material [file JCMM-25-7901-s001.zip › jcmm16713-sup-0001-Data/Figure 2/Fig 2 Migration/Fig 2-Control 48h migration.tif]
